# Differential effects of alkyl gallates on quorum sensing in *Pseudomonas aeruginosa*

**DOI:** 10.1038/s41598-019-44236-w

**Published:** 2019-05-23

**Authors:** Bomin Kim, Ji-Su ParK, Ha-Young Choi, Jin-Hwan Kwak, Won-Gon Kim

**Affiliations:** 1 Superbacteria Research Center, Korea Research Institute of Bioscience and Biotechnology, Yusong, Daejeon, 305–806 Korea; 2 Department of Bio-Molecular Science, KRIBB School of Bioscience, Korea University of Science and Technology (UST), Yusong, Daejeon 305–806 Korea; 3 School of Life Science, Handong Global University, Pohang, Kyungbuk 37554 Korea

**Keywords:** Antibiotics, Biofilms

## Abstract

Virulence factors and biofilms constitute attractive targets for the prevention of infections caused by multidrug-resistant bacteria. Among alkyl gallates, propyl gallate (PG) and octyl gallate (OG) are used as food preservatives. Here we found that alkyl gallates differentially affect virulence, biofilm formation, and quorum sensing (QS) in *Pseudomonas aeruginosa*. Ethyl gallate (EG), PG, and butyl gallate (BG) inhibited biofilm formation and virulence factors including elastase, pyocyanin, and rhamnolipid, in *P. aeruginosa* without affecting cell viability by antagonizing the QS receptors LasR and RhlR. PG exhibited the most potent activity. Interestingly, hexyl gallate (HG) inhibited the production of rhamnolipid and pyocyanin but did not affect elastase production or biofilm formation. Notably, OG inhibited the production of rhamnolipid and pyocyanin but stimulated elastase production and biofilm formation. Analysis of QS signaling molecule production and QS gene expression suggested that HG inhibited RhlR, while OG activated LasR but inhibited PqsR. This mechanism was confirmed using QS mutants. Additionally, PG prevented the virulence of *P. aeruginosa* in *Caenorhabditis elegans* and a mouse model. This is the first report of the differential effects of alkyl gallates on QS systems and PG has great potential as an inhibitor of the virulence and biofilm formation of *P. aeruginosa*.

## Introduction

Quorum sensing (QS) is a bacterial communication system that uses small diffusible signal molecules known as autoinducers. Bacterial population behaviors regulated by QS include bioluminescence, sporulation, biofilm formation, virulence factor production, and antibiotic resistance^1–3^. Many pathogenic bacteria utilize QS for the production of virulence factors and biofilms. Thus, the inhibition of QS is considered a new promising target for the control of bacterial infections. Traditional strategies for the prevention and treatment of bacterial infections are based on the use of antibacterial compounds that kill bacteria or inhibit the growth of bacteria. These strategies have resulted in substantial stress on target bacteria, causing the rapid growth of resistant populations^4^. Virulence factors and biofilm are not essential for bacterial survival. Thus, interference with bacterial virulence factors and biofilms is a new therapeutic strategy because this method produces less selection pressure for the development of resistance than traditional strategies^5^.

*Pseudomonas aeruginosa* is a Gram-negative bacteria that is highly resistant to existing antibiotics and cause many opportunistic and nosocomial infections. In particular, chronic lung infections with *P. aeruginosa* are the major causes of mortality in cystic fibrosis (CF) patients^6^. *P. aeruginosa* has been shown to form biofilms in the CF lung, which increase bacterial resistance to antibiotics^7^, and also produce several virulence factors including elastase, rhamnolipid, and pyocyanin. Thus, the inhibition of virulence factor production and biofilm formation may be highly attractive for the prevention and treatment of *P. aeruginosa* infections^8^.

QS in *P*. *aeruginosa* is tightly regulated by three main QS systems organized in a hierarchical manner^3^. LasR-LasI and RhlR-RhlI use acyl homoserine lactones (AHLs) as signaling molecules, while PqsR-PqsABCD uses 2-alkyl-4-quinolones. LasI and RhlI, the AHL synthases, synthesize N-(3-oxododecanoyl)-l-homoserine lactone (OdDHL) and N-butanoyl homoserine lactone (BHL), respectively, whereas PqsABCD, the quinolone synthase, produces 2-heptyl-3-hydroxy-4(1 H) quinolone (PQS). When activated by OdDHL, the LasR-OdDHL complex activates the transcription of *lasI* and *lasR*, which leads to autoinduction. The LasR-OdDHL complex also initiates the expression of the *rhl* and *pqs* QS systems and directs the gene expression of biofilms and virulence factors such as elastase^3,9^. The RhlR-BHL complex, in turn, activates the expression of *rhlI* and activates many QS-dependent virulence factors, such as rhamnolipid and pyocyanin. The PqsR-PQS complex also activates the gene cascades associated with the PQS system and virulence factors such as pyocyanin and rhamonolipids^10^. Recently, IQS (2-(2-hydroxyphenyl) thiazole-4-carbaldehyde), was discovered as a fourth QS signal molecule. The IQS system is tightly controlled by *las* under normal conditions, but take over the functions of the central *las* system under phosphate depletion stress conditions^11^.

Gallic acid (GA) and alkyl gallates are found in several natural and industrial products. For example, GA and propyl gallate (PG) are abundant in green tea and octyl gallate (OG) has been found in the medicinal plant *Terminalia bellerica*^12^. Notably, PG and OG have been approved for use as antioxidant additives in the food and pharmaceutical industries for over 60 years because these compounds exhibit significantly low toxicity both *in vitro* and *in vivo*^13–15^. PG and OG are used at concentrations of up to 0.015% (0.7 mM) and 0.4% (1.4 mM), respectively, in foods^13,16^. To date, in addition to their antioxidant activity, alkyl gallates have been reported to exhibit various other biological activities and the chain lengths of alkyl gallates differentially affects the pharmacological activity of these compounds depending on the specific pharmacological activity^17,18^. Alkyl gallates were reported to have antimicrobial activity against Gram-positive bacteria and some fungi, but almost no antibacterial activity against Gram-negative bacteria^19–21^. Very recently, phenolic compounds, including methyl gallate (MG), have been reported to exhibit antibiofilm activity against *Streptococcus mutans* and anti-QS activity against *P. aeruginosa*, but the antivirulence activity of alkyl gallates, especially PG and OG, has not been studied to date^22–24^.

While screening for an inhibitor of *P. aeruginosa* virulent factors from a microbial metabolite library, we identified gallate-like compounds. Here, we report the differential effects of alkyl gallates on virulence factors and biofilms, the *in vivo* antivirulence activity of PG against *P. aeruginosa*, and the differential effects of alkyl gallates on QS systems.

## Results

### Effect of alkyl gallates on the production of virulence factors

**Figure 1:**
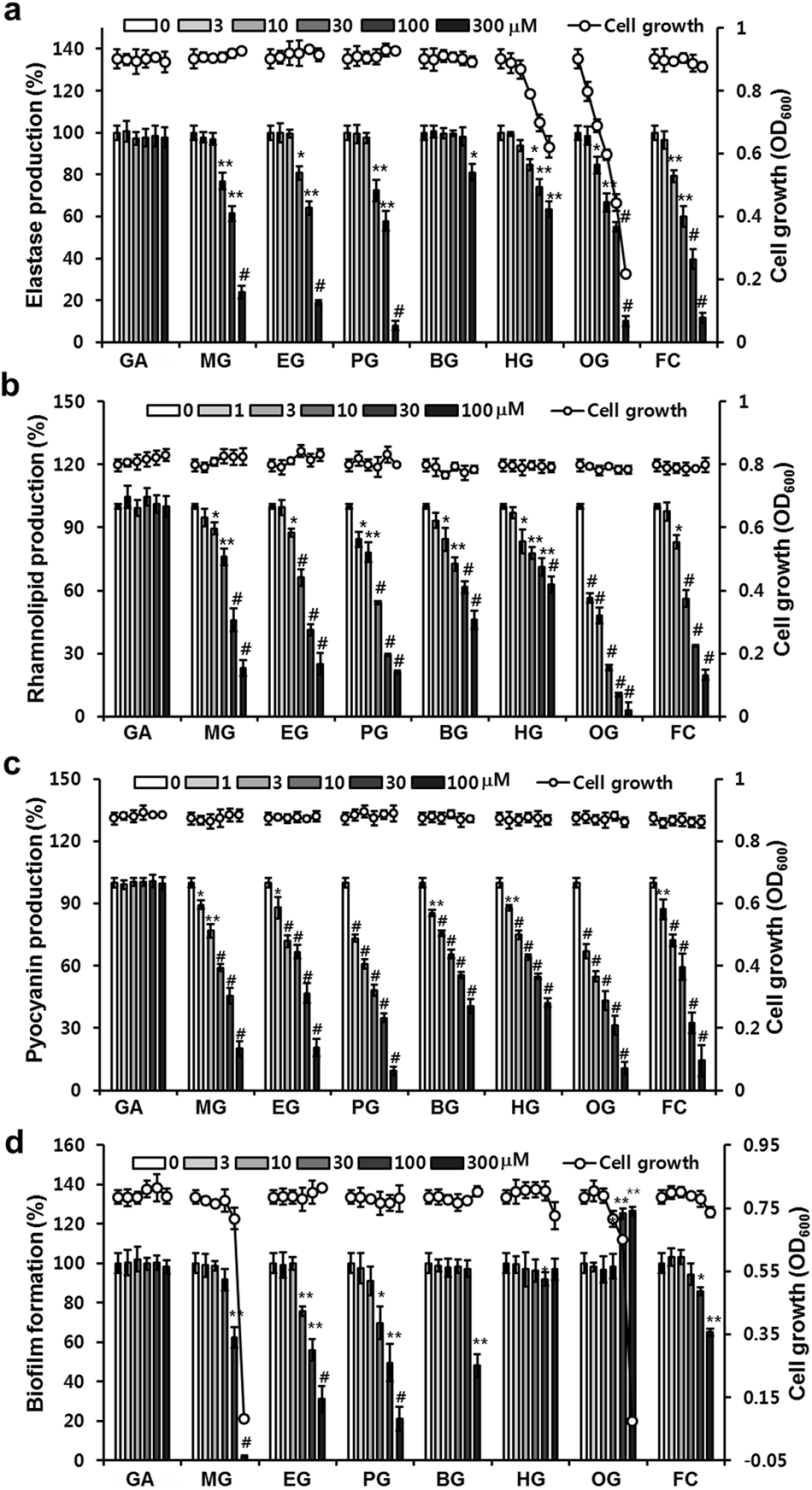
Effects of alkyl gallates on *P. aeruginosa* virulence factor production, biofilm formation, and growth. (**a**,**b**,**c**) Effects of alkyl gallates on *P. aeruginosa* virulence factor production and cell growth. After PAO1 cells were grown in LB medium in the presence of different concentrations of alkyl gallates for 24 h, cell density was measured at 600 nm and elastase activity and pyocyanin and rhamnolipid in the culture supernatants were then determined. (**d**) Effects of alkyl gallates on *P. aeruginosa* biofilm formation and cell growth. PAO1 biofilms were grown in the presence of alkyl gallates for 9 h, followed by the measurement of planktonic cell density at 600 nm and the biofilm cells attached to the well surfaces using crystal violet staining. Three independent experiments were done in triplicate, and the mean ± SD values are presented in each bar. *P < 0.01; **P < 0.001; ^#^*P* < 0.0001 versus untreated cells.

The effects of alkyl gallates on the production of elastase, pyocyanin, and rhamnolipid by *P. aeruginosa* PAO1 cells were evaluated. Six alkyl gallates (MG, ethyl gallate (EG), PG, butyl gallate (BG), hexyl gallate (HG), and OG) and GA were tested. The antibacterial activity of the alkyl gallates was also examined against each virulence factor using an optical-density-based assay. MG, EG, and PG inhibited elastase production in PAO1 cells in a dose-dependent manner without affecting cell viability (Figs 1a and S1). PG exhibited the strongest inhibition of elastase production. The elastase activity of the PAO1 cells was significantly inhibited by 27.5% and 92.1% in the presence of 30 and 300 μM PG for 24 h, respectively, compared with that of untreated cells. BG weakly inhibited elastase production by 20.2% at only 300 μM. However, HG and OG exhibited antibacterial activity within the range required for inhibition of elastase production (Fig. 1a). The specific inhibitory activity (enzyme activity per unit of cell mass) of HG and OG on elastase production suggested that OG enhanced elastase production whereas HG did not affect elastase production (Fig. S2). As a control, furanone C-30 (FC) exhibited similar inhibition as PG, while GA exhibited no inhibition.

In the rhamnolipid and pyocyanin assays, all the alkyl gallates tested dose-dependently reduced the production of rhamnolipid and pyocyanin in PAO1 cells without inhibiting cell growth, whereas GA exhibited no activity (Fig. 1b,c). MG, EG, and PG exhibited increasing inhibition of rhamnolipid and pyocyanin production with increasing alkyl chain length. Pyocyanin production by PAO1 cells was significantly inhibited by 26.8%, 51.9% and 90.7% in the presence of 1, 10, and 100 μM PG for 24 h, respectively, compared with the production in untreated cells (Fig. 1b). Similarly, rhamnolipid production by PAO1 cells in the presence of 1, 10, and 100 μM PG was also inhibited by 15.7%, 46.0% and 78.9%, respectively. However, BG and HG exhibited weak inhibitory activity against the production of rhamnolipid and pyocyanin. OG again strongly inhibited the production of rhamnolipid and pyocyanin. These results indicated that the alkyl group is critical for the inhibitory activity against virulence factor production, regardless of alkyl chain length.

### Effect of alkyl gallates on biofilm formation

It is reported that rhamnolipid and pyocyanin play a role in biofilm formation of *P. aeruginosa* PAO1^25,26^. Because the alkyl gallates differentially inhibited the production of rhamnolipid and pyocyanin, biofilm formation assays were carried out by staining the biofilm biomass to determine whether alkyl gallates affect the development of growing *P. aeruginosa* PAO1 biofilms. The effects of alkyl gallates on the growth of planktonic cells were also examined via an optical-density-based assay. MG exhibited biofilm inhibition activity at 100 and 300 μM but also inhibited the growth of planktonic cells at the same concentrations, suggesting that the antibiofilm activity of MG might be due to its antibacterial activity (Fig. 1d). EG and PG exhibited dose-dependent inhibition of biofilm formation at 30–300 μM without affecting planktonic cell viability, while GA did not exhibit the same effect. BG exhibited weak antibiofilm activity at only 300 μM, similar to the result of the elastase production assay. However, HG did not inhibit biofilm formation even at 300 μM. Interestingly, OG enhanced biofilm formation at 100 and 300 μM with a decrease in planktonic cell density, suggesting that OG stimulated biofilm formation. To confirm the enhancement of biofilm formation by OG, the effects of OG on the production of extracellular polymeric substances (EPS) in biofilms were investigated. Indeed, OG enhanced the production of extracellular polysaccharides and proteins in biofilms, while PG inhibited EPS production and HG did not affect EPS production, which was consistent with the effects of these compounds on biofilm formation (Fig. S3). These different (inhibitory, non-inhibitory, and enhancing) effects of PG, HG, and OG on biofilm formation were consistent with the effects of these compounds on elastase production, suggesting that PG, HG, and OG might differentially regulate the Las system.

The antibiofilm activity of PG was confirmed by confocal laser scanning microscopy. Static grown of PAO1 cells for 6 h led to the formation of a biofilm with a thickness of 18.9 ± 1.34 μm. The treatment of 100 μM PG to the cells, however, resulted in a substantial decrease in the biofilm thickness to 10.8 ± 1.25 μm, and the live bacterial cell density also decreased (Fig. S4).

The antivirulence and antibiofilm effects of the alkyl gallates against clinical isolates were investigated (Figs S5, S6). The alkyl gallates showed the differential effects on virulence factor production and biofilm formation in six clinical *P. aeruginosa* isolates including drug-resistant strains, similar to in the PAO1 strain.

### Inhibitory effects of alkyl gallates on QS

The effects of alkyl gallates on QS receptors were carried out by an AHL-based *in vitro* QS competition assay using two reporter strains, namely, *Chromobacterium violaceum* CV026 and *Agrobacterium tumefaciens* NT1^27^, as reported previously^28^. Briefly, *A. tumefaciens* NT1 carries the *lacZ* reporter gene fused with the TraR receptor gene. The TraR receptor can detect AHLs with a long carbon chain, such as OdDHL, resulting in the production of a cyan pigment^29^. The *C. violaceum* CV026 strain contains the CviR receptor, which can sense AHLs with a short carbon chain, such as BHL, leading to the synthesis of a purple pigment called violacein^30^. The TraR and CviR receptors are homologs of the LasR and RhlR receptors, respectively. Thus, competitive binding assays of the LasR and RhlR receptors is conducted using these reporter strains^31,32^.

**Figure 2:**
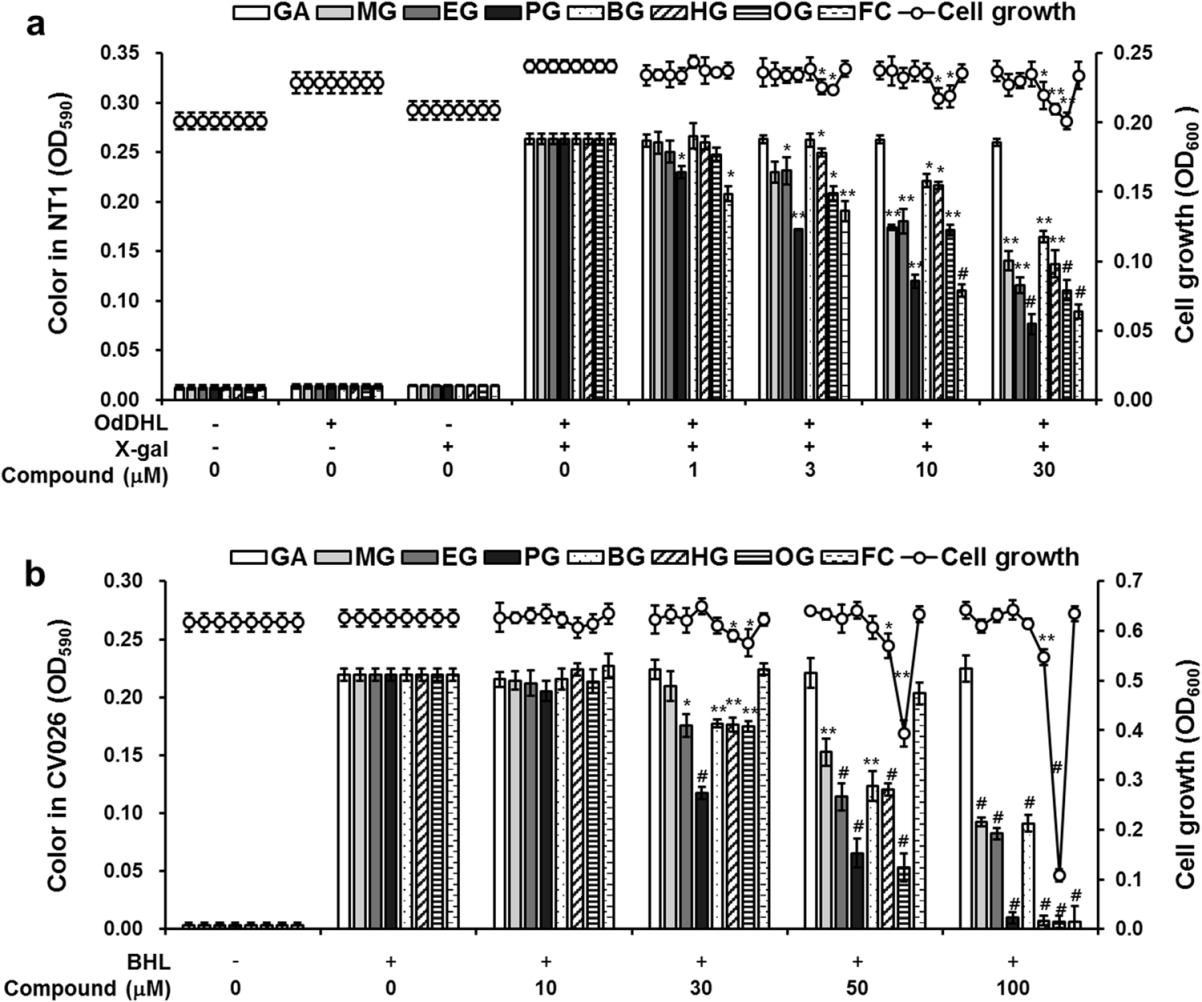
Effects of alkyl gallates on QS. QS competition with BHL and OdDHL was performed using the reporter strains *A. tumefaciens* NT1 and *C. violaceum* CV026, respectively. After 1 μM OdDHL and 500 μM BHL were added to NT1 and CV026 cultures, respectively, containing various concentrations of alkyl gallates and incubated for 24 h, measurements of cell growth at 600 nm and changes in color were done. (**a**) Alkyl gallate–OdDHL competition. (**b**) Alkyl gallate–BHL competition. Data are expressed as the mean ± SD values of three independent experiments performed in triplicate. *P < 0.01; **P < 0.001; ^#^*P* < 0.0001 versus untreated cells.

**Figure 3:**
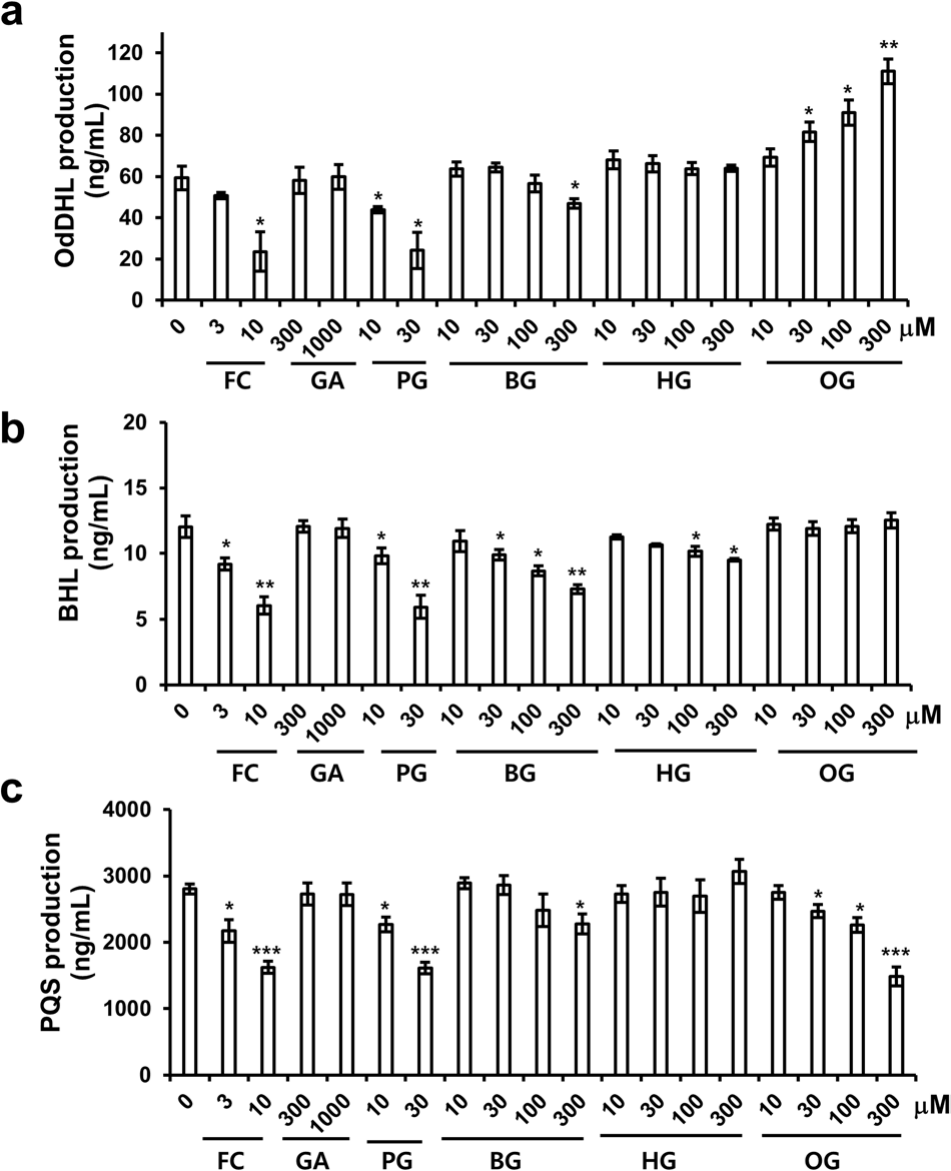
Effects of alkyl gallates on QS signaling molecule production. PAO1 cells were cultured in LB medium containing alkyl gallates or furanone C-30 (FC) for 12 h. The three main QS molecules, namely, OdDHL (**a**), BHL (**b**), and PQS (**c**), were extracted from the culture supernatants and quantitatively analyzed by LC-MS/MS. Data are representative of three independent experiments performed in triplicate and expressed as the mean ± SD values in each bar. *P < 0.01; **P < 0.001; ***P < 0.0001 versus DMSO treatment.

Pigment was produced in NT1 cultures only when OdDHL was added, as expected (Fig. 2a). MG, EG, and PG reduced pigment production at 3–30 μM without affecting cell viability. PG exhibited the most potent activity. BG weakly inhibited pigment production at 10–30 μM. HG and OG inhibited pigment production at 10−30 μM, but also exhibited antibacterial activity, suggesting that the inhibition of pigment production might be due to the antibacterial activity of these compounds. Similarly, the production of the violacein pigment was inhibited by MG, EG, PG, and BG in CV026 cultures without any effect on cell viability (Fig. 2b). However, HG and OG also exhibited antibacterial activity against CV026 cells. The inhibition potency (BG < MG < EG < PG) of MG, EG, PG, and BG against pigment production in both reporter strains was consistent with the inhibition of virulence factor production and biofilm formation. Additionally, consistent with the absence of any effects on virulent factors and biofilm formation, GA exhibited no inhibition of pigment production in either reporter strain, as expected. This finding indicated that MG, EG, PG, and BG antagonized the binding of OdDHL and BHL to the cognate receptors LasR and RhlR, respectively. However, the effects of HG and OG on QS receptors could not be determined using these reporter strains due to the antibacterial activity of these compounds. FC, a well-known QS inhibitor, inhibited the binding of OdDHL to TraR at 1–30 μM (Fig. 3a) but antagonized CviR at only 100 μM (Fig. 2b).

### Effects of alkyl gallates on QS signaling molecule production and QS gene expression

To examine whether HG and OG inhibit QS, the effects of these compounds on the production of QS signaling molecules were investigated in the culture conditions used for rhamnolipid and pyocyanin assays, in which these compounds did not exhibit antibacterial activity. The production of OdDHL, BHL, and PQS molecules in PAO1 cells was quantitatively determined by LC-MS/MS (Fig. 3). The untreated PAO1 cultures in the 12-h culture supernatants provided yields of 59.4 ± 5.7 ng/mL, 12.0 ± 0.8 ng/mL, and 2803 ± 77.7 ng/mL for OdDHL, BHL, and PQS, respectively. Interestingly, HG inhibited only BHL production at 100 and 300 μM without affecting OdDHL and PQS production. Notably, OG increased OdDHL production in a dose-dependent manner but decreased PQS production at 30–300 μM without affecting on BHL production. PG reduced the production of the three QS molecules at 10 and 30 μM, similar to the positive control FC, and BG exhibited weak inhibition, which was consistent with the weak inhibition of virulence factor production. GA had no activity, as expected.

**Figure 4:**
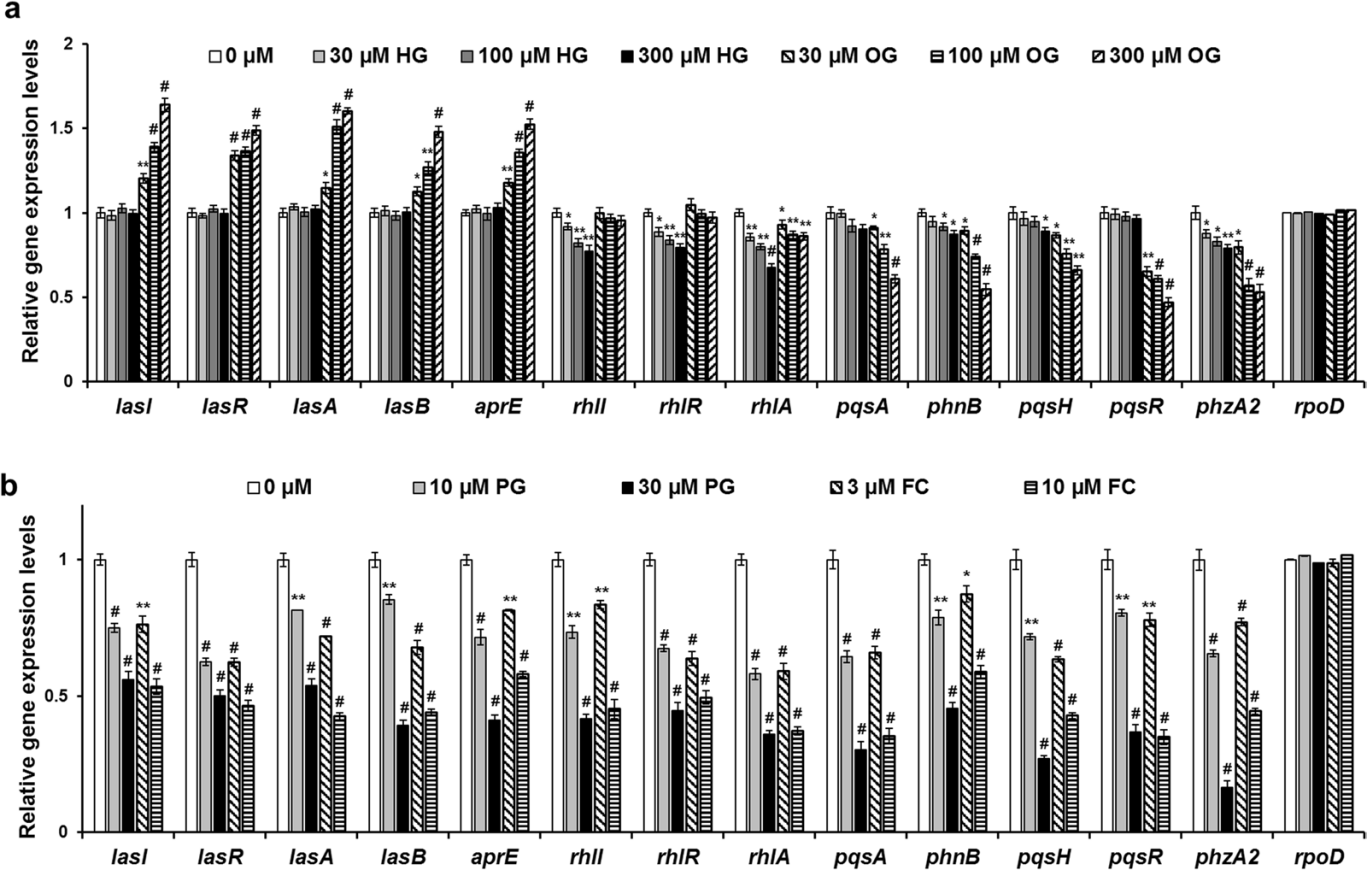
Effects of alkyl gallates on QS gene expression. PAO1 cells were cultured in LB medium in the presence of alkyl gallates for 12 h. The effects of the alkyl gallates on QS gene expression were measured by RT-qPCR. The experiment shown is representative of three independent experiments performed in triplicate and the mean ± SD values are presented in each bar. *P < 0.01; **P < 0.001; ^#^*P* < 0.0001 versus DMSO treatment.

The effects of alkyl gallates on QS gene expression were measured by real-time quantitative PCR (RT-qPCR). The transcription of QS-regulatory genes (*lasI, IasR, rhlI, rhlR, pqsA, phnB, pqsH*, and *pqsR*) and key QS-controlled genes (*lasA, lasB, aprE, rhlB*, and *phzE1*) in PAO1 strains was investigated (Fig. 4). Consistent with the selective and weak inhibition of BHL production by HG, this compound weakly repressed the transcription of the *rhlI* and *rhlR* genes and the genes associated with the biosynthesis of rhamnolipid and pyocyanin (*rhlB* and *phzE1*, respectively) with almost no effect on the other genes. In contrast, OG dramatically activated the transcription of *las* genes, while repressing the transcription of the *pqs, rhlB*, and *phzE1* genes, with almost no effect on the transcription of the *rhlI* and *rhlR* genes (Fig. 4a). Notably, the OG-induced increase in the transcription of the elastase genes *lasA* and *lasB* confirmed the enhancement of elastase production by OG (Figs 1a and S2). PG dose-dependently repressed the transcriptional levels of the QS-related genes at 10 and 30 μM like FC, similar to the positive control FC, as expected (Fig. 4b). BG exhibited weak inhibition, while GA had no inhibitory activity (Fig. S7), as expected. These results suggested that HG inhibited the Rhl system, while OG activated the Las system but inhibited the Pqs system. Additionally, the results demonstrated the antagonistic effects of PG on the QS receptors LasR and RhlR.

The effects of the alkyl gallates on QS signaling molecule production and QS gene expression in clinical isolates were investigated. The alkyl gallates showed the differential effects on QS signaling molecule production (Fig. S8) and QS gene expression (Figs S9, S10) in the clinical *P. aeruginosa* isolates, similar to in the PAO1 strain.

Additionally, the effects of the alkyl gallates on IQS signaling system were investigated by RT-PCR of the biosynthesis gene of IQS (*ambB*). As a control experiment, in low phosphate *Pseudomonas* medium supplemented with 4 mM K_2_HPO_4_ in which IQS is regulated by *las*^11^, the transcription level of *ambB* was significantly reduced in the LasI and LasR-deficient strains compared with the levels in the wild-type strain (PA14) and the alkyl gallates inhibited the transcription levels of *ambB* in the three strains as expected (Fig. S11). In contrast, in low phosphate *Pseudomonas* medium in which IQS is not controlled by *las*^11^, the transcription of *ambB* was not reduced in the Las-deficient strains compared with the levels in the wild-type strain as expected. The alkyl gallates did not inhibit the transcription of *ambB* in the three strains (Fig. S12). These results indicated that the alkyl gallates did not affect IQS signaling.

### Agonistic effects of OG on LasR

**Figure 5:**
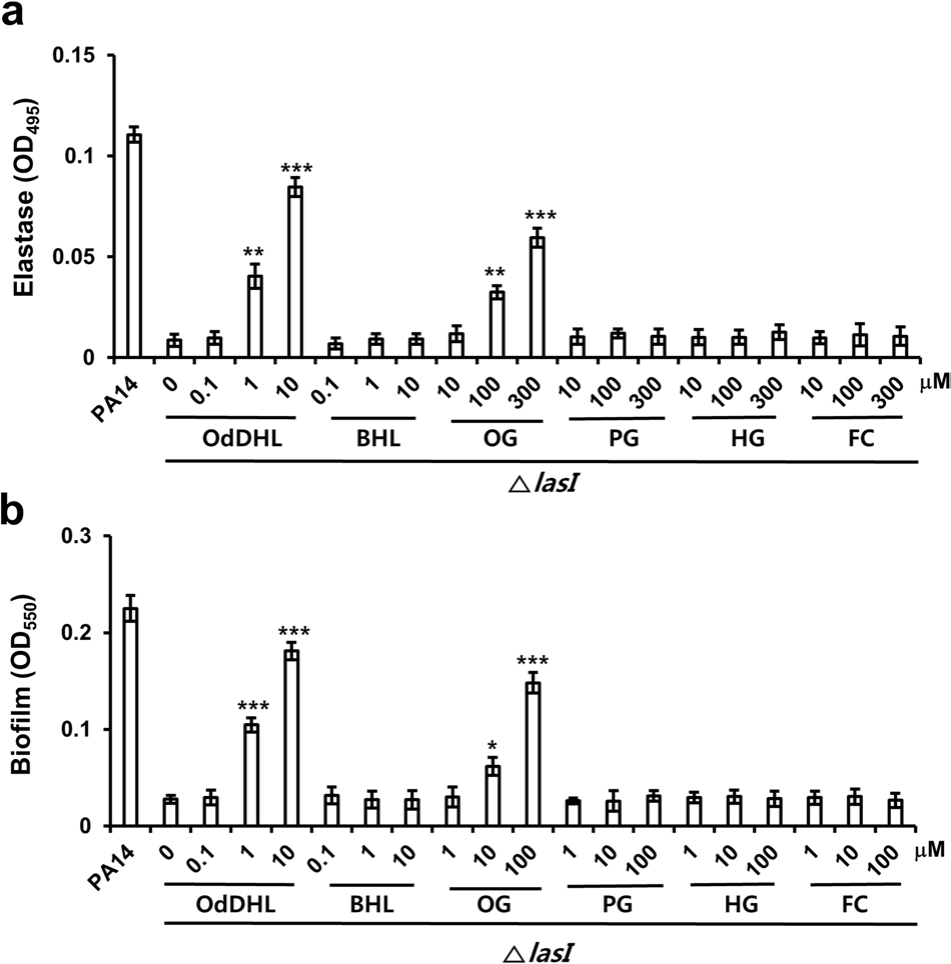
Effects of OG on elastase production and biofilm formation in the Δ*lasI* mutant. The ΔlasI mutant of PA14 was cultured in a medium containing different concentrations of alkyl gallates and QS ligands for elastase production and biofilm formation. Data are representative of three independent experiments performed in triplicate and expressed as the mean ± SD values in each bar. *P < 0.01; **P < 0.001; ***P < 0.0001 versus DMSO treatment.

The LasR-OdDHL complex regulates elastase production and biofilm formation^10^. To investigate how OG activates the Las system, the agonistic effects of OG on LasR in *P. aeruginosa* were tested by analyzing the effect of OG on elastase production and biofilm formation in a Δ*lasI* mutant^33^ of the PA14 strain, lacking the ability to produce the LasR ligand OdDHL. First, the effects of the alkyl gallates on virulence factor production, biofilm formation, QS signal molecule production, and QS-related genes were confirmed in wild-type PA14 cells (Figs S13, S14, and S15). Elastase production and biofilm formation were reduced by 91.9% and 87.6%, respectively, in the Δ*lasI* mutant compared with the levels in the wild-type strain (Fig. 5a,b). The addition of OdDHL as a positive control increased elastase production and biofilm formation in a dose-dependent manner at 1 and 10 μM in the Δ*lasI* mutant, while BHL, as a negative control, did not affect elastase production and biofilm formation. Similarly, elastase production in the Δ*lasI* mutant in the presence of 100 and 300 μM OG increased 3.6 and 6.6 times, respectively, in a dose-dependent manner and biofilm formation also increased 2.2 and 5.3 times at 10 and 100 μM OG, respectively, compared with the levels in untreated cells. In contrast, FC, PG, and HG did not exhibit these agonistic effects at all. This result indicated the agonistic effect of OG on LasR. This agonistic effect of OG on LasR was verified by the expression of Las-related genes in the Δ*lasI* mutant (Fig. S16). OG initiated the transcription of *lasR*, *lasA*, *lasB* and *aprE* in the Δ*lasI* mutant, similar to OdDHL, while the other compounds did not affect the transcription of these genes. These results indicated the agonistic effect of OG on LasR.

### Effects of HG and OG on RhlR and PqsR

To determine whether HG and OG inhibited virulence factor production via inhibition of the Rhl and Pqs QS systems, respectively, the effects of exogenous QS ligands on the HG- or OG-induced reduction of pyocyanin and rhamnolipid were investigated in PA14 cells. As shown in Fig. S17, exogenous supplementation of BHL (0.1–10 μM) but not PQS reversed the inhibitory activity of HG (100 μM) on the production of rhamnolipid and pyocyanin in a dose-dependent manner (Fig. S17a,c). In contrast, exogenous supplementation of PQS (0.1–10 μM) but not BHL reversed the inhibitory activity of OG (10 μM) on the production of rhamnolipid and pyocyanin in a dose-dependent manner (Fig. S17b,d). As controls, FC- and PG-induced inhibition of pyocyanin and rhamnolipid were reversed by supplementation with both PQS and BHL. This result confirmed that HG and OG inhibit virulence factor production by inhibiting the Rhl and Pqs systems, respectively.

**Figure 6:**
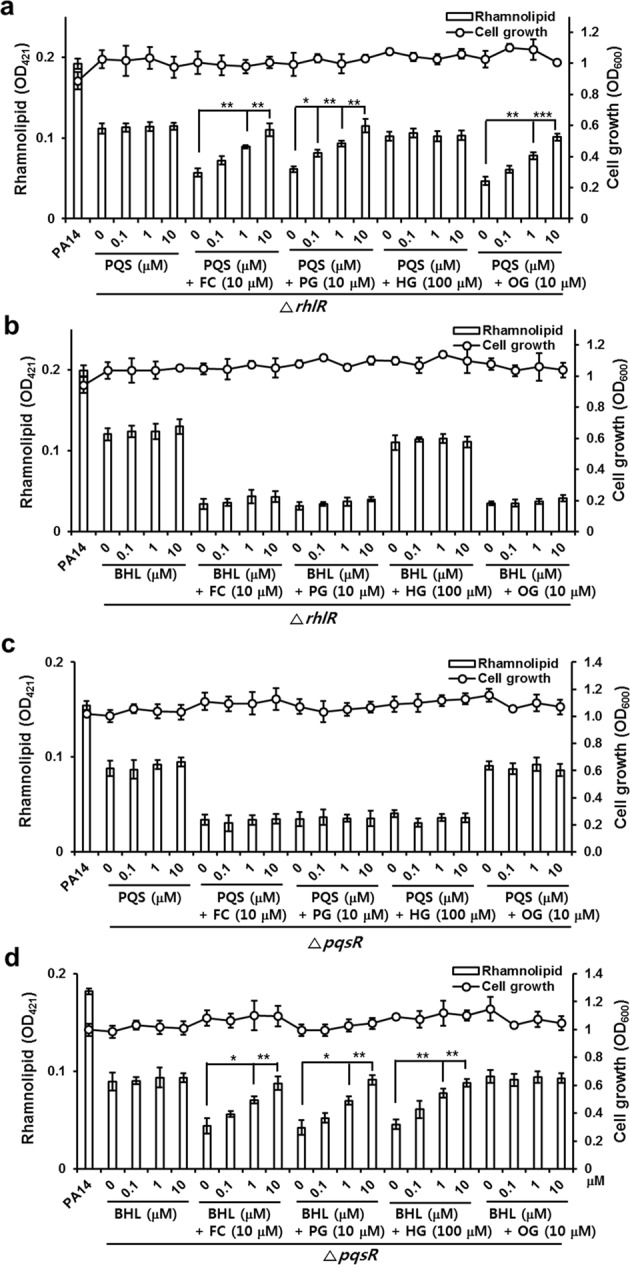
Effects of exogenous QS ligands on HG- and OG-induced inhibition of rhamnolipid production in Δ*rhlR* and Δ*pqsR* mutants. (**a**,**b**) Rhamnolipid production in the Δ*rhlR* mutant (**a**,**b**) and Δ*pqsR* mutant (**c**,**d**) cultured with alkyl gallates in the presence or absence of different concentrations of PQS (**a**,**c**) or BHL (**b**,**d**) for 18 h. Data are representative of three independent experiments performed in triplicate and expressed as the mean ± SD values in each bar. *P < 0.01; **P < 0.001; ***P < 0.0001 versus DMSO treatment.

To demonstrate that HG and OG inhibited the production of rhamnolipid and pyocyanin by antagonizing RhlR and PqsR, respectively, the effects of these compounds on the production of rhamnolipid and pyocyanin in *rhlR* and *pqsR* mutants^33^ were investigated. HG barely inhibited the production of rhamnolipid and pyocyanin in the *rhlR* mutant, and this inhibition was not affected by exogenous PQS and BHL (Figs 6a,b, S18a,b). However, HG inhibited rhamnolipid production in the *pqsR* mutant, and this inhibition was reversed by exogenous BHL but not PQS (Fig. 6c,d). This result indicated that HG inhibited the production of rhamnolipid and pyocyanin by inhibiting RhlR. Similarly, OG did not inhibit rhamnolipid production in the *pqsR* mutant, and this effect was not influenced by exogenous PQS and BHL (Fig. 6c,d). However, OG inhibited rhamnolipid production in the *rhlR* mutant, and this inhibition was reversed by exogenous PQS but not BHL (Fig. 6a,b). This result indicated that OG inhibited the production of rhamnolipid and pyocyanin by inhibiting PqsR. The effects on pyocyanin production in the *pqsR* mutant could not be studied because almost no pyocyanin was produced in the *pqsR* mutant. As controls, FC and PG inhibited the virulence factor production in both the *rhlR* and *pqsR* mutants, and this inhibition was reversed by exogenous PQS and BHL (Figs 6 and S18), respectively, confirming that FC and PG inhibited the production of rhamnolipid and pyocyanin by inhibiting RhlR and PqsR.

### Antivirulence effects of PG in a *C. elegans* infection model

**Figure 7:**
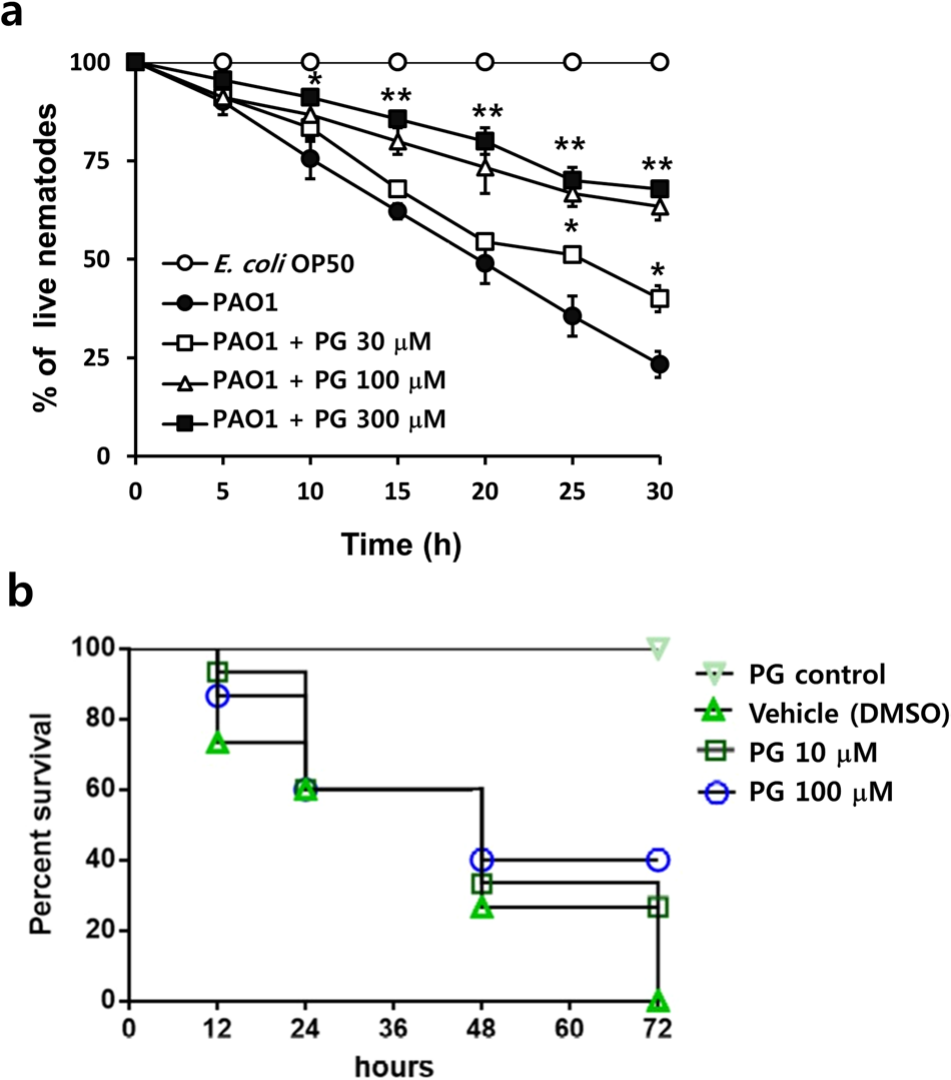
*In vivo* antivirulence activity of PG. (**a**) Antivirulence effects of PG in a *C. elegans* infection model. Thirty worms were introduced on lawns of *E. coli* OP50 (open circles) or PAO1 (filled circles) on plates in the presence of different concentrations of PG. Live nematodes were counted every 5 h for 30 h. Data are presented as the mean ± SD values of three independent experiments performed in triplicate. *P < 0.01; **P < 0.001 versus untreated cells. The presence of PG (30~300 μM) significantly protected *C. elegans* from killing. (**b**) Antivirulence effects of PG in a murine airway infection model. Mouse survival rate was calculated following infection with PAO1 without or with PG (10 and 100 μM). Fifteen mice were used in each group.

The effects of PG on *in vivo* virulence of *P. aeruginosa* were investigated using a *C. elegans* fast-kill infection assay. When *C. elegans* were fed with *P. aeruginosa* PAO1, 75% of the nematodes died after 30 h (Fig. 7a). In the presence of PG, the death of the nematodes was substantially reduced in a dose-dependent manner. The percentage of nematode death significantly decreased after PG treatment at 30–300 μM. These results clearly indicated that PG protected *C. elegans* against infection with *P. aeruginosa*. Other alkyl gallates also reduced the virulence of *P. aeruginosa* in *C. elegans*, while GA did not, consistent with their *in vitro* antivirulence effects (Fig. S19).

### Antivirulence effects of PG in a murine airway infection model

To further assess the protective effect of PG against PAO1 infection, a murine airway infection model was employed. A total of fifteen mice were used in each group for a survival experiment. PAO1-infected mice began to expire at 12 h postinfection, and completely expired at 72 h. However, six of the fifteen mice treated with PG were alive until the termination of the experiment at 72 h (Fig. 7b). This result strongly suggests that PG attenuated the virulence of *P. aeruginosa* in the mouse airway.

## Discussion

EG, PG, and BG inhibited virulence factor production and biofilm formation in the *P. aeruginosa* PAO1 and PA14 strains by antagonizing the QS receptors LasR and RhlR without affecting cell viability. Additionally, the effects of these compounds on QS signaling molecule production and QS gene expression were verified. PG exhibited the most potent inhibition in both PAO1 and PA14 cells, while BG exhibited the weakest activity. Interestingly, HG inhibited the production of rhamnolipid and pyocyanin, but did not affect elastase production or biofilm formation. Notably, OG inhibited the production of rhamnolipid and pyocyanin, but stimulated elastase production and biofilm formation. Because the antagonizing effects of HG and OG on QS receptors could not be determined using the reporter strains due to the antibacterial activity of these compounds, their effects on QS signaling molecule production and QS gene expression were investigated. HG exhibited selective inhibition of BHL molecule production and *rhl* gene expression. OG increased OdDHL molecule production and *las* gene expression but decreased PQS molecule production and *pqs* gene expression. These results, together with the results of the QS ligand supplementation experiments, suggested that HG might selectively inhibit RhlR, while OG activates LasR and inhibits PqsR; this mechanism was verified using QS mutants of PA14. This is the first report of the anti-QS effects of PG, selective inhibition of RhlR by HG, and differential effects of OG on LasR and PqsR. The highest levels (300 μM) of PG and OG used in all experiments were at least two-times lower than those (0.7 mM and 1.4 mM, respectively) used in food.

The three QS systems in *P. aeruginosa* are organized hierarchically, with LasR at the top of the signaling cascade^3,9^, and are also interconnected^34^. When activated by OdDHL, the LasR-OdDHL complex triggers the onset of both the *rhl* and *pqs* systems by activating the transcription of *rhlI*, *rhlR*, *pqsR*, and *pqsH*. PqsR (also known as MvfR) is the transcriptional regulator of the PQS biosynthesis operon *pqs*ABCD, and PqsH completes the final step of PQS synthesis. The PqsR-PQS complex enhances the transcription of *rhlI*, thus influencing the overall expression of the *rhl* QS system^35,36^. However, the RhlR-BHL complex represses PQS signal production by inhibiting the expression of *pqsR* and *pqs*ABCD^37,38^. Thus, it has been suggested that the ratio of OdDHL to BHL determines the activation of PQS synthesis^38^. LasR-OdDHL induces the production of virulence factors, such as the LasA and LasB elastases and alkaline protease, and biofilm formation^10^. RhlR-BHL and PqsR-PQS direct gene expression of several virulence factors, such as rhamnolipid, pyocyanin, and hydrogen cyanide^35^. In this study, similar to FC, PG inhibited virulence factor production (elastase, rhamnolipid, and pyocyanin) and biofilm formation by antagonizing the QS receptors LasR and RhlR. Additionally, as shown by using the *rhlR* mutant (Figs 6a,b, S18a,b), FC and PG also inhibited Pqs-dependent production of rhamnolipid and pyocyanin, which occurred via inhibition of LasR and subsequent downregulation of the Pqs system. HG inhibited only Rhl-dependent production of rhamnolipid and pyocyanin by inhibiting RhlR, without affecting elastase production and biofilm formation which are regulated by the Las system. Because the Rhl system negatively regulates the expression of *pqs*ABCD and subsequent PQS production^37,38^, HG, an inhibitor of RhlR, is expected to increase the production of PQS molecules and the expression of Pqs-related genes. Because HG did not affect the production of PQS molecules or the expression of Pqs-related genes at 10–300 μM, the effect of higher concentrations of HG on the Pqs system was tested. Indeed, HG enhanced the production of PQS molecules and the expression of Pqs-related genes at 500 μM and higher concentration (Fig. S20a,b). Additionally, pyocyanin production increased at the higher concentrations of HG, and this effect was also reversed by exogenous BHL (Fig. S20c). This result suggested that BHL strongly down-regulates the Pqs system such that strong inhibition of Rhl QS is necessary for the BHL-induced repression of Pqs QS to be alleviated. This is the first report demonstrating BHL-induced repression of Pqs QS using a RhlR inhibitor. On the other hand, OG activated LasR and inhibited PqsR. Las-OdDHL has been reported to positively regulate the Pqs system. Because OG inhibited the expression of Pqs-related genes, PQS production, and subsequent Pqs-dependent production of rhamnolipid and pyocyanin, OG is hypothesized to inhibit PqsR more potently than activated LasR.

Alkyl gallates have been reported to exhibit various biological activities, including cardiovascular protection^39^, antihyperglyceric^12^, antibacterial^19,20,40^, antifungal^18,21^, antiviral^41^, anticancer^15^, and anti-inflammatory activities^42^. The chain lengths of alkyl gallates differentially affects the pharmacological activity of these compounds depending on the specific pharmacological activity^17,18,43^. These compounds act as antioxidants in a variety of ways including differential reacting with radicals^15^. Alkyl gallates exhibit antibacterial activity against Gram-positive bacteria by disrupting membrane permeability, which occurs due to the membrane binding properties of the hydrophobic alkyl chains of these compounds^17^. The antibacterial activity of alkyl gallates on *S. aureus* and *B. subtilis* increased with alkyl chain length for side chains of 3–10 carbons^40^. However, the correlation between the chain length of the alkyl gallates and membrane binding activity differs depending on the microorganism^17^. Although the correlation is high for *S. aureus* and *B. subtilis*, intermediate correlation was observed for *S. mutans* and low correlation was observed for fungi such as *Trichophyton rubrum* and *Microsporum gypseum*^18^. Alkyl gallates have almost no antibacterial activity against Gram-negative bacteria. Alkyl gallates with long chains (C8 and C12)^22^ and MG^44^ were reported to have antibacterial activity against *Salmonella* strains but not against other Gram-negative bacteria including *P. aeruginosa*, *E. coli* etc.

The antibacterial activity of antimicrobial compounds varies with inoculum size, growth medium, incubation conditions and endpoint determination method^45^. Because each assay for biofilm formation and virulence factor production used different media and incubation conditions, the antibacterial activity of the alkyl gallates in each assay was measured in parallel to determine whether the inhibitory activity of these compounds on biofilm formation and virulence factor production is due to growth inhibition activity. Indeed, the alkyl gallates tested exhibited differences in antibacterial activity among the various assays performed in this study. Interestingly, PG had no antibacterial activity in all the assays, even at 2000 μM, but alkyl gallates with shorter or longer alkyl chains than PG exhibited antibacterial activity against *P. aeruginosa* in this study (Table S2).

PG, propyl 3,4,5-trihydroxybenzoate, is an ester formed by the condensation of GA and propanol. Since 1948, PG has been added to foods containing oils and fats to protect fats, oils, and fat-containing food from oxidation^13,14^. PG also exhibits potential anti-inflammatory effects by inhibiting LPS-induced iNOS expression and nitric oxide production^42^. PG has been reported to weakly inhibit the growth of mainly Gram-positive bacteria such as *Staphylococcus aureus*, but not Gram-negative bacteria such as *P. aeruginosa*^22–24^. The antibiofilm and antivirulence activity of PG against Gram-negative bacteria is reported for the first time in this study.

In conclusion, among the alkyl gallates tested, PG most potently reduces *P. aeruginosa* biofilm formation and virulence factor production without affecting cell viability by antagonizing LasR and RhlR. Additionally, PG exhibited *in vivo* antivirulence activity in *C. elegans* and mouse models. However, OG activated the Las system and the subsequent production of elastase and biofilm formation, although this compound inhibited the Pqs system and the subsequent production of rhamnolipid and pyocyanin. Thus, although both PG and OG have been used as food additives, only PG presents the possibility for use as a safe material to treat biofilm-associated infections problem in healthcare facilities or as a medication for treating drug-resistant *P. aeruginosa* infections.

## Methods

### Elastase assay

Elastase activity in the supernatant of *P. aeruginosa* PAO1 and PA14 was determined using an elastin-Congo red assay as previously described^46^. Overnight cultures of *P. aeruginosa* were grown in LB medium at 37 °C with shaking. The cultures were then diluted 1:100 into fresh LB medium and dispensed at 0.1 mL/well in a 96-well microtiter plate. Test compounds dissolved in DMSO were treated to the cultures for 24 h. After cell viability was determined by measuring the optical density (OD) at 600 nm, the cultures were centrifuged at 4000 rpm for 10 min and the elastase activity of the culture supernatants was measured.

### Pyocyanin, rhamnolipid, and biofilm assays

Pyocyanin^47^ and rhamnolipid^48^ production and biofilm formation^49^ in *P. aeruginosa* PAO1 and PA14 were measured as previously described (Supporting Information). After incubation for 24 h, cell viability was assayed by measuring the OD at 600 nm, and pyocyanin and rhamnolipid were then extracted and determined at 520 and 421 nm, respectively. Biofilm cells were measured using crystal violet staining.

### QS competition assay

An AHL-based QS competition assay was carried out using two reporter strains, namely, *A. tumefaciens* NT1 and *C. violaceum* CV026^27^. Overnight cultures of each strain were grown in LB medium at 30 °C. One mL of the 20-fold-diluted overnight culture was transferred into a 15-mL conical tube and 5 μL of X-gal (20 g/L) were added to *A. tumefaciens* NT1. Ten microliters of 100 μM OdDHL (Sigma) and 50 mM BHL (Cayman, Ann Arbor, MI, USA) were then added to *A. tumefaciens* NT1 and *C. violaceum* CV026, respectively. Finally, 10 μL of each test compound dissolved in DMSO were treated to the cultures, followed by incubation at 30 °C for 48 h. After cell viability was assayed by measuring the OD at 600 nm using a microtiter ELISA reader, color changes in *C. violaceum* CV026 and *A. tumefaciens* NT1 were measured at 590 nm and 545 nm, respectively.

### Measurement of QS signaling molecules and QS gene expression

The effects of alkyl gallates on QS signaling molecule production^50^ and QS gene expression^51^ were determined as previously described. The culture method for *P. aeruginosa* PAO1 and PA14 was the same as that used for rhamnolipid and pyocyanin assays. For the measurement of three QS signaling molecules, 3 mL of the culture supernatants were extracted with 3 mL of ethyl acetate acidified with 0.1% acetic acid, and the organic layer was then evaporated and measured by LC-MS/MS in MRM mode (Supporting Information). For the determination of QS gene expression, total RNA was extracted using TRIzol reagent (Invitrogen), followed by synthesis of cDNA for RT-qPCR detection of the expression of target genes and measurement by RT-qPCR using a Bio-Rad CFX-96 real− time PCR system (Bio-Rad, Hercules, CA, USA) with the primers listed in Table S1 (Supporting Information). mRNA expression was normalized to the expression of the endogenous *rpoD* gene.

### C. elegans life span assay

A *P. aeruginosa*-*C. elegans* infection assay was conducted as previously described^52^. *C. elegans* nematodes were grown on NGM plates containing an ample lawn of *E. coli* OP50 as a food source at 20 °C for 48 h. Killing and control plates were prepared by spreading 5 μL of overnight cultures of PAO1 and OP50 cells, respectively, on a 35-mm petri plate with 4 mL of PGS agar. Test compounds dissolved in DMSO were treated to the killing plates. The plates were then incubated at 37 °C for 24 h to make a bacterial lawn and then shifted to 23 °C for 24 h. Thirty L4-stage worms were placed on each plates and incubated at room temperature. Nematodes were counted for survival every 5 h for 30 h.

### Murine airway infection

Twenty 5-week-old BALB/C inbred female mice (Orient, Korea) were infected with 2.5 × 10^7^ PAO1 cells. Bacteria was precultured for 20 h and diluted 5-fold in fresh MHA medium or in MHA supplemented with 10 or 100 μM PG. After shaking incubation for 7 h, the PAO1 cultures were centrifuged, and the pellets were washed with saline, and then resuspended to 5 × 10^8^ cfu/mL. Anesthesia was induced prior to infection by intraperitoneal injection with Zoletil 50 (50 g/L) + Rompun (23.32 g/L) at 0.006 mL/10 g + 0.004 mL/10 g. Mice were infected with a dose of 2.5 × 10^7^ cfu/mouse in 50 μL PBS via the intranasal inhalation (n = 15 for each group). Infected mice were observed for survival for 72 h.

### Statistical analysis

Data are presented as mean ± standard deviation (SD) values. *P-*values for testing statistical differences between measurements were estimated by the unpaired Student’s *t*-test (Excel software, Microsoft, Redmond, WA, USA). A *p*-value < 0.05 was considered statistically significant.

### Ethics

All animal experiments were approved by the Committee on the Ethics of Animal Experiments of Handong Global University (South Korea) (protocol #HGU-20170921-006). All experiments were performed in accordance with the approved guidelines of the Institutional Ethical Committee, adhered to Guide for the Care and Use of Laboratory Animals of National Research Council (USA).

## Supplementary information

Supplementary Information

## References

[1] Ng WL, Bassler BL (2009). Bacterial quorum-sensing network architectures. Annu Rev Genet.

[2] Rutherford S. T., Bassler B. L. (2012). Bacterial Quorum Sensing: Its Role in Virulence and Possibilities for Its Control. Cold Spring Harbor Perspectives in Medicine.

[3] Papenfort K, Bassler BL (2016). Quorum sensing signal-response systems in Gram-negative bacteria. Nat Rev Microbiol.

[4] Werner G, Strommenger B, Witte W (2008). Acquired vancomycin resistance in clinically relevant pathogens. Future Microbiol.

[5] Rasko DA, Sperandio V (2010). Anti-virulence strategies to combat bacteria-mediated disease. Nat Rev Drug Discov.

[6] Finnan S, Morrissey JP, O’Gara F, Boyd EF (2004). Genome diversity of Pseudomonas aeruginosa isolates from cystic fibrosis patients and the hospital environment. J. Clin. Microbiol..

[7] Davies D (2003). Understanding biofilm resistance to antibacterial agents. Nat Rev Drug Discov.

[8] Bjarnsholt T, Ciofu O, Molin S, Givskov M, Hoiby N (2013). Applying insights from biofilm biology to drug development - can a new approach be developed?. Nat Rev Drug Discov.

[9] Lee J, Zhang L (2015). The hierarchy quorum sensing network in Pseudomonas aeruginosa. Protein Cell.

[10] Jimenez PN (2012). The multiple signaling systems regulating virulence in Pseudomonas aeruginosa. Microbiol Mol Biol Rev.

[11] Lee J (2013). A cell-cell communication signal integrates quorum sensing and stress response. Nat Chem Biol.

[12] Eler GJ (2015). n-Octyl gallate as inhibitor of pyruvate carboxylation and lactate gluconeogenesis. J Biochem Mol Toxicol.

[13] Final report on the amended safety assessment of Propyl Gallate. *Int J Toxicol* **26**(Suppl 3), 89–118, 10.1080/10915810701663176 (2007). 10.1080/10915810701663176 18080874

[14] Dolatabadi JEN, Kashanian S (2010). A review on DNA interaction with synthetic phenolic food additives. Food Research International.

[15] Jara JA (2014). Antiproliferative and uncoupling effects of delocalized, lipophilic, cationic gallic acid derivatives on cancer cell lines. Validation *in vivo* in singenic mice. J. Med. Chem..

[16] EFSA. Scientific opinion on the re-evaluation of octyl gallate (E 311) as a food additive. *EFSA Journal* **13**, 4248, 10.2903/j.efsa.2015.4248 (2015).

[17] Takai E, Hirano A, Shiraki K (2011). Effects of alkyl chain length of gallate on self-association and membrane binding. J. Biochem..

[18] Leal PC (2009). Relation between lipophilicity of alkyl gallates and antifungal activity against yeasts and filamentous fungi. Bioorg. Med. Chem. Lett..

[19] Kubo I, Xiao P, Fujita K (2002). Anti-MRSA activity of alkyl gallates. Bioorg. Med. Chem. Lett..

[20] Krol E (2015). Antibacterial activity of alkyl gallates is a combination of direct targeting of FtsZ and permeabilization of bacterial membranes. Front Microbiol.

[21] Ito S, Nakagawa Y, Yazawa S, Sasaki Y, Yajima S (2014). Antifungal activity of alkyl gallates against plant pathogenic fungi. Bioorg. Med. Chem. Lett..

[22] Kubo I, Fujita K, Nihei K (2002). Anti-Salmonella activity of alkyl gallates. J. Agric. Food. Chem..

[23] Zheng CJ, Oh HW, Kim WG (2010). Potent anticariogenic activity of Aceriphyllum rossii and its components, aceriphyllic acid A and 3-oxoolean-12-en-27-oic acid. J. Food Sci..

[24] Rua J (2010). Screening of phenolic antioxidants for their inhibitory activity against foodborne Staphylococcus aureus strains. Foodborne Pathog Dis.

[25] Das T (2015). Phenazine virulence factor binding to extracellular DNA is important for Pseudomonas aeruginosa biofilm formation. Sci Rep.

[26] Van Gennip M (2009). Inactivation of the rhlA gene in Pseudomonas aeruginosa prevents rhamnolipid production, disabling the protection against polymorphonuclear leukocytes. APMIS.

[27] Park SY (2006). N-acylhomoserine lactonase producing Rhodococcus spp. with different AHL-degrading activities. FEMS Microbiol. Lett..

[28] Kim B, Park JS, Choi HY, Yoon SS, Kim WG (2018). Terrein is an inhibitor of quorum sensing and c-di-GMP in Pseudomonas aeruginosa: a connection between quorum sensing and c-di-GMP. Sci Rep.

[29] Zhang L, Murphy PJ, Kerr A, Tate ME (1993). Agrobacterium conjugation and gene regulation by N-acyl-L-homoserine lactones. Nature.

[30] McClean KH (1997). Quorum sensing and Chromobacterium violaceum: exploitation of violacein production and inhibition for the detection of N-acylhomoserine lactones. Microbiology.

[31] Kim HS, Lee SH, Byun Y, Park HD (2015). 6-Gingerol reduces Pseudomonas aeruginosa biofilm formation and virulence via quorum sensing inhibition. Sci Rep.

[32] Srivastava A, Singh BN, Deepak D, Rawat AK, Singh BR (2015). Colostrum hexasaccharide, a novel Staphylococcus aureus quorum-sensing inhibitor. Antimicrob. Agents Chemother..

[33] Park SY, Heo YJ, Choi YS, Deziel E, Cho YH (2005). Conserved virulence factors of Pseudomonas aeruginosa are required for killing Bacillus subtilis. J Microbiol.

[34] Higgins S (2018). Differential Regulation of the Phenazine Biosynthetic Operons by Quorum Sensing in Pseudomonas aeruginosa PAO1-N. Front Cell Infect Microbiol.

[35] Diggle SP (2003). The Pseudomonas aeruginosa quinolone signal molecule overcomes the cell density-dependency of the quorum sensing hierarchy, regulates rhl-dependent genes at the onset of stationary phase and can be produced in the absence of LasR. Mol. Microbiol..

[36] McKnight SL, Iglewski BH, Pesci EC (2000). The Pseudomonas quinolone signal regulates rhl quorum sensing in Pseudomonas aeruginosa. J. Bacteriol..

[37] Brouwer S (2014). The PqsR and RhlR transcriptional regulators determine the level of Pseudomonas quinolone signal synthesis in Pseudomonas aeruginosa by producing two different pqsABCDE mRNA isoforms. J. Bacteriol..

[38] McGrath S, Wade DS, Pesci EC (2004). Dueling quorum sensing systems in Pseudomonas aeruginosa control the production of the Pseudomonas quinolone signal (PQS). FEMS Microbiol. Lett..

[39] Kosuru Rekha Yamini, Roy Amrita, Das Sujoy K., Bera Soumen (2017). Gallic Acid and Gallates in Human Health and Disease: Do Mitochondria Hold the Key to Success?. Molecular Nutrition & Food Research.

[40] Kubo I, Fujita K, Nihei K, Nihei A (2004). Antibacterial activity of akyl gallates against Bacillus subtilis. J. Agric. Food. Chem..

[41] Uozaki M (2007). Antiviral effect of octyl gallate against DNA and RNA viruses. Antiviral Res.

[42] Jeon W, Park SJ, Kim BC (2017). n-Propyl gallate suppresses lipopolysaccharide-induced inducible nitric oxide synthase activation through protein kinase Cdelta-mediated up-regulation of heme oxygenase-1 in RAW264.7 macrophages. Eur. J. Pharmacol..

[43] Barla F (2009). Inhibitive effects of alkyl gallates on hyaluronidase and collagenase. Biosci Biotechnol Biochem.

[44] Choi JG (2014). Methyl gallate from Galla rhois successfully controls clinical isolates of Salmonella infection in both *in vitro* and *in vivo* systems. PLoS One.

[45] Balouiri M, Sadiki M, Ibnsouda SK (2016). Methods for *in vitro* evaluating antimicrobial activity: A review. J Pharm Anal.

[46] Rust L, Messing CR, Iglewski BH (1994). Elastase assays. Methods Enzymol..

[47] Essar DW, Eberly L, Hadero A, Crawford IP (1990). Identification and characterization of genes for a second anthranilate synthase in Pseudomonas aeruginosa: interchangeability of the two anthranilate synthases and evolutionary implications. J. Bacteriol..

[48] Boles BR, Thoendel M, Singh PK (2005). Rhamnolipids mediate detachment of Pseudomonas aeruginosa from biofilms. Mol. Microbiol..

[49] O’Toole, G. A. Microtiter dish biofilm formation assay. *J Vis Exp*, 10.3791/2437 (2011). 10.3791/2437 PMC3182663 21307833

[50] Luo J (2017). Baicalin inhibits biofilm formation, attenuates the quorum sensing-controlled virulence and enhances Pseudomonas aeruginosa clearance in a mouse peritoneal implant infection model. PLoS One.

[51] Gi M (2014). A drug-repositioning screening identifies pentetic acid as a potential therapeutic agent for suppressing the elastase-mediated virulence of Pseudomonas aeruginosa. Antimicrob. Agents Chemother..

[52] O’Loughlin CT (2013). A quorum-sensing inhibitor blocks Pseudomonas aeruginosa virulence and biofilm formation. Proc Natl Acad Sci USA.

