# Supplementary material for: Differential effects of alkyl gallates on quorum sensing in Pseudomonas aeruginosa
Source: Sci Rep. 2019 May 23;9:7741. doi: 10.1038/s41598-019-44236-w (PMC6533263; doi:10.1038/s41598-019-44236-w)
Supplement: Supplementary file 1 — Supplementary Information [file 41598_2019_44236_MOESM1_ESM.pdf]

Supporting Information

Differential effects of alkyl gallates on quorum sensing in *Pseudomonas aeruginosa*

Bomin Kim[1], Ji-Su ParK[1], Ha-Young Choi[1], Jin-Hwan Kwak[2], and Won-Gon Kim[1]*

[1]Superbacteria Research Center, Korea Research Institute of Bioscience and Biotechnology, Yusong, Daejeon 305-806, Korea; [2]School of Life Science, Handong Global University, Pohang, Kyungbuk 37554, Korea. Correspondence and requests for materials should be addressed to W.-G. K. (e-mail: wgkim@kribb.re.kr).

Pyocyanin and rhamnolipid assay. For both pyocyanin and rhamnolipid production, after overnight cultures of *P. aeruginosa* PAO1 were diluted 100–fold in LB medium, 5 mL of culture was dispensed into 50 mL conical tubes, treated with test compounds dissolved in DMSO, and incubated at 220 rpm at 37°C for 24 h. The cultures were then centrifuged at 12,000 rpm at 4°C for 10 min. For pyocyanin assay, 5 mL of supernatant was mixed with 3 mL of chloroform and 2 mL of 0.2 N HCl was added to the chloroform fraction. The resultant aqueous fraction was measured at 520 nm by a microplate reader. For rhamonolipid assay, 500 μL supernatant was mixed with 500 μL diethyl ether. The ether fraction was evaporated to dryness and dissolved in 500 μL deionized water. 100 μL of the water extract was mixed with 900 μL of Orcinol solution (0.19% Orcinol (Sigma) in 53% H_2SO_4). The mixture was boiled for 30 min, cooled at room temperature for 15 min, and then measured at 421 nm by a microplate reader.

Biofilm assay. Overnight cultures of *P. aeruginosa* PAO1 were diluted 100–fold in M63 medium and the dilutions were dispensed at 0.1 mL/well in a 96-well polystyrene microplate. Test compounds or DMSO as a negative control were added to the wells. After incubation at 37°C for 9 h without agitation, the unattached cells and media were removed and the cells forming the biofilm, which remained attached to the well's surface, were stained using 120 μL of 0.1% crystal violet for 10 min. The bound crystal violet was solubilized with 150 μL of 30% acetic acid in water for 15 min. The OD of the eluted crystal violet was measured at 550 nm by a microplate reader.

Confocal laser scanning microscopy. Biofilms of PAO1 were grown on 15 mm^2 glass coverslips (Matsunami Glass Ind., Ltd. Japan). Sterile coverslips were positioned vertically in 24-well plates. Cultures were grown for 6 h without agitation at 37°C. Coverslips were then washed and stained with SYTO9/propidium iodide according to the manufacturer′s instructions of the L13152 LIVE/DEAD Baclight bacterial viability kit (Invitrogen Molecular Probes, USA). After staining for 15 min in the dark, biofilms were washed with sterile phosphate-buffered saline (PBS) to remove the planktonic dyes and bacteria, and then biofilms were

visualized by excitation with an argon laser at 488 nm (emission: 515 nm) and 543 nm (emission: 600 nm) under a Confocal laser scanning microscope (Carl Zeiss LSM800, Jena, Germany).

Quantification of QS signaling molecules by LC-MS/MS

The samples were subjected to an HPLC system (Luna C18(2), 100 × 2.0 mm, 3 μm, Phenomenex, Torrance, CA, USA) connected to a QTrap 3200 with a TurboIon Spray source (AB SCIEX, Singapore). The column was maintained at 20°C with a flow rate of 0.4 mL/min and a gradient of acetonitrile in 0.1% (v/v) aqueous formic acid; 0-10 min from 70% to 100%. MRM was performed by selecting the two mass ions set specifically for the selected analytes to detect the transition from parent ion to product ion, i.e., *m/z* 298.211 > 197.200 for OdDHL, *m/z* 172.181 > 71.000 for BHL, and *m/z* 260.244 > 188.100 for PQS (Sigma). For analysis of OdDHL, BHL, and PQS, the TurboIon Spray source-dependent parameters were optimized to the following values: 10 psi curtain gas, high collision gas, 5500 V ion spray voltage, 400°C temperature, and 12 psi ion source gas. The compound-dependent parameters for OdDHL, BHL, and PQS were optimized to the following values: 19, 17, and 41 eV collision energy; 5, 8, and 12 V entrance potential; 276, 31, and 71 V declustering potential; and 18, 10, and 16 V collision cell exit potential, respectively.

RT-qPCR of QS-regulated genes

cDNA was synthesized from 2 μg of RNA mixed with 1 μg of random primers (Promega C1181) and RNase-free water (Sigma W4502) in a total of 13.37 μL and incubated for 5 min at 70°C. Thereafter, 1 μL of M-MLV reverse transcriptase (Promega M170 200 U/μL), 5 μL 5 × M-MLV buffer (Promega M531), 5 μL of deoxynucleoside triphosphates (Enzynomics N001S, 2 mM), and 0.63 μL of RNasin® Ribonuclease Inhibitors (Promega N251, 2500 U/μL) were added and incubated for 60 min at 42°C. The cDNA samples were used for RT-qPCR detection

of the expression of target genes. RT-qPCR was performed using the Bio-Rad CFX-96 real time system (Bio-Rad, Hercules, CA, USA) with the primers listed in Table S1. Amplification and expression were carried out in a total volume of 20 μL containing 10 μL SYBR Premix Ex Taq™ (Takara, Shiga, Japan), 1 μL each of the forward and reverse primers (5 μM) of target genes, 2 μL template cDNA, and 6 μL RNase-free water. The cycling parameters were as follows: initial activation at 95℃ for 30 s; 40 cycles at 95℃ for 5 s, 60℃ for 30 s, and melting curve analysis at 95℃ for 15 s, 60℃ for 5 s and 95℃ for 5 s. mRNA expression was normalized using the endogenous *rpoD* gene.

Table S1. Primers used for quantitative RT-PCR

Gene and primer type	PCR primer sequence (5' to 3')
lasI	
Forward	TTCAAGGAGCGCAAAGGCTG
Reverse	GTTCTTCAGCATGTAGGGGC
lasR	
Forward	TCTGGGAACCGTCCATCTAC
Reverse	GACCGACTCCATGAAACGGT
lasA	
Forward	GACGACCTGTTCCTCTACGG
Reverse	GCTCCAGGTATTCGCTCTTG
lasB	
Forward	CCGCAAGACCGAGAATGACA
Reverse	CTTCCCACTGATCGAGCACT
aprE	
Forward	ATGTACATCGTGCCCAACAG
Reverse	GGTCTTGCTCTGGTTGAAGG
rhlI	
Forward	CTTCATCGAGAAGCTGGGCT
Reverse	AGGTAGGCGAAGACGTCCTT
rhlR	
Forward	TGCATGATCGAGTTGCTG
Reverse	GTGCTCTCGGAGATGCTCA
rhlA	
Forward	GCGCGAAAGTCTGTTGGTAT
Reverse	CAGCACCACGTTGAAATGTT
pqsA	
Forward	CCACTCCGCTGGACGACAAC
Reverse	GCAGCATGTGCGAGGGAATC
phnB	
Forward	CACTCGCTGGTGGTCAGTC
Reverse	AGAGTAGAGCGTTCTCCAGCA
pqsH	
Forward	ATGTCTACGCGACCCTGAAG
Reverse	AACTCCTCGAGGTCGTTGTG
pqsR	
Forward	CTTCGCCTGATCCCTTACAT
Reverse	TGAAATCGTCGAGCAGTACG
phzA2	
Forward	AACCACTTCTGGGTCGAGTG
Reverse	TCGAGTTCGAAGGAATGGAT
ambB	
Forward	ATCACTACGCCTTCGACGAT
Reverse	ATCGAGCAGCGTCTTCAGTT
rpoD	
Forward	GGGGATCAACGTATTCGAGA
Reverse	GGTACCCATTTCACGCATGT

Table S2. Antibacterial activity of alkyl gallates in virulence factor and biofilm assays.

Assays		GA	MG	EG	PG	BG	HG	OG
Biofilm	MIC_{50}	>3000	254.8 ± 45.0	1556.0 ± 222.1	>3000	>3000	656.4 ± 26.8	163.0 ± 8.1
Rhamnolipid and pyocycanin	MIC_{50}	>2000	>2000	>2000	>2000	>2000	1078 ±26.2	417.3 ± 0.7
	MIC	>2000	>2000	>2000	>2000	>2000	2000	2000
Elastase	MIC_{50}	>4000	1444.7 ± 62.1	1847.7 ± 170.1	>4000	1020.9 ± 299.0	397 ± 13.3	217.4 ± 18.7
	MIC	>4000	4000	4000	>4000	4000	4000	2000

Antibacterial activities (MIC_{50} or MIC) in each assay were evaluated. Three independent experiments were performed in triplicate, and the mean ± SD values are displayed.

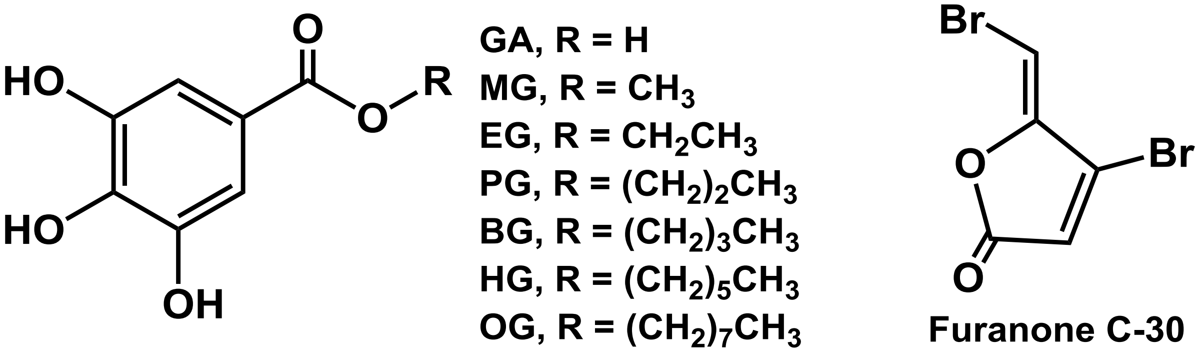

Supplementary Figure S1. Chemical structures of alkyl gallates and related compounds.

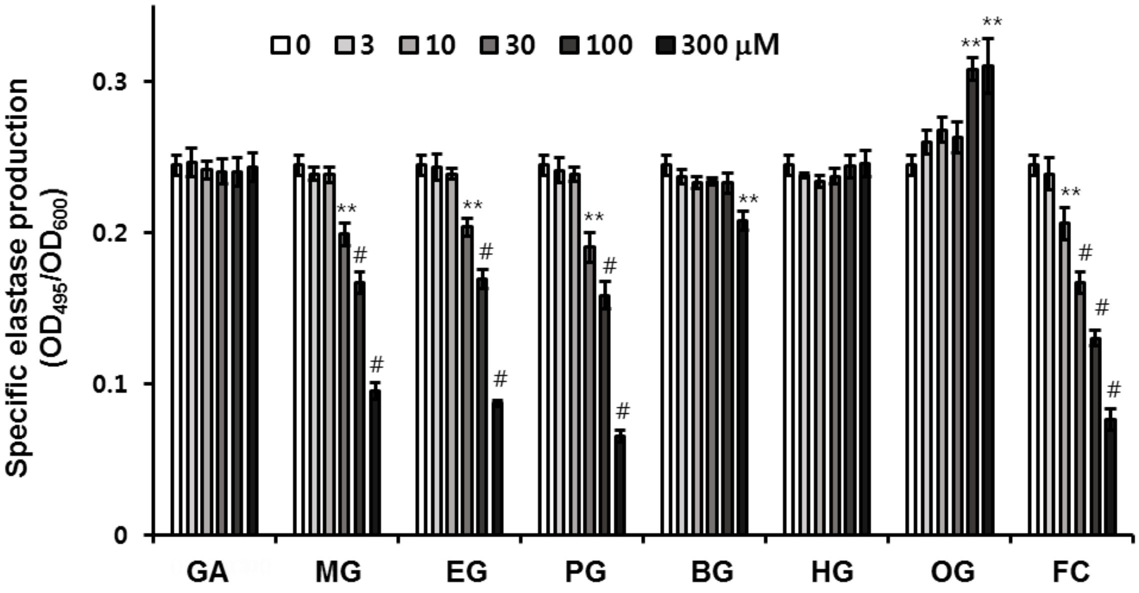

Supplementary Figure S2. Effects of alkyl gallates on specific elastase activity in *P. aeruginosa* PAO1. PAO1 cells were grown in LB medium containing different concentrations of alkyl gallates for 24 h, followed by the measurement of cell density at 600 nm and elastase activity at 495 nm in the culture supernatants. The specific elastase activity was measured as the absorbance of the supernatant (OD_{495}) divided by the OD_{600} of the culture normalized to 1 mL of supernatant. Three independent experiments were performed in triplicate, and the mean ± standard deviation (SD) values are displayed in each bar. *, $P < 0.01$; **, $P < 0.001$; $^{\#}$, $P < 0.0001$ versus untreated cells.

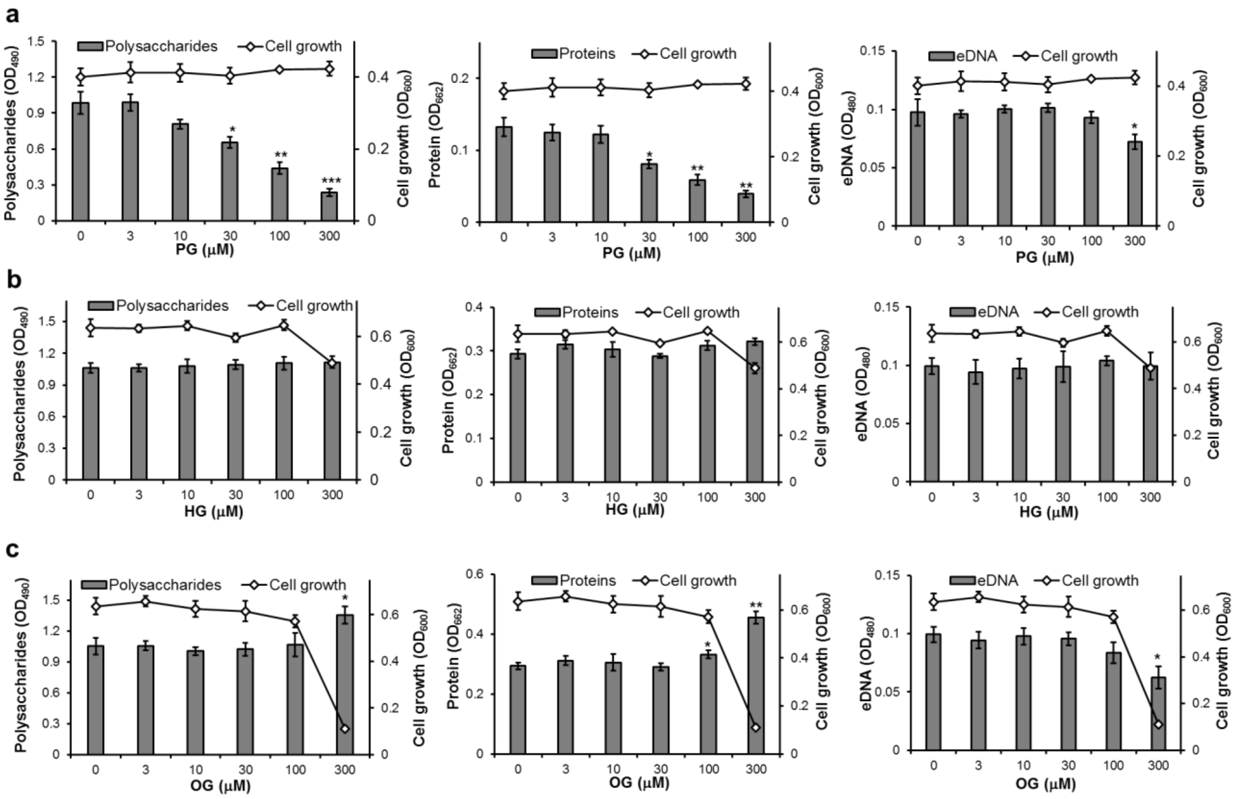

Supplementary Figure S3. Effect of OG on extracellular polymer substance production in biofilm formation of *P. aeruginosa* PAO1. *P. aeruginosa* biofilms formed in the presence of different concentration of PG (a), HG (b), and OG (c) for 9 h. Three extracellular polymer substances, polysaccharides, protein, and extracellular DNA (eDNA), were extracted and assessed from the biofilms. Planktonic cells density were measured at 600 nm. Three independent experiments were performed in triplicate, and the mean ± standard deviation (SD) values are displayed in each bar. *, $P < 0.01$; **, $P < 0.001$; ***, $P < 0.0001$ versus untreated cells.

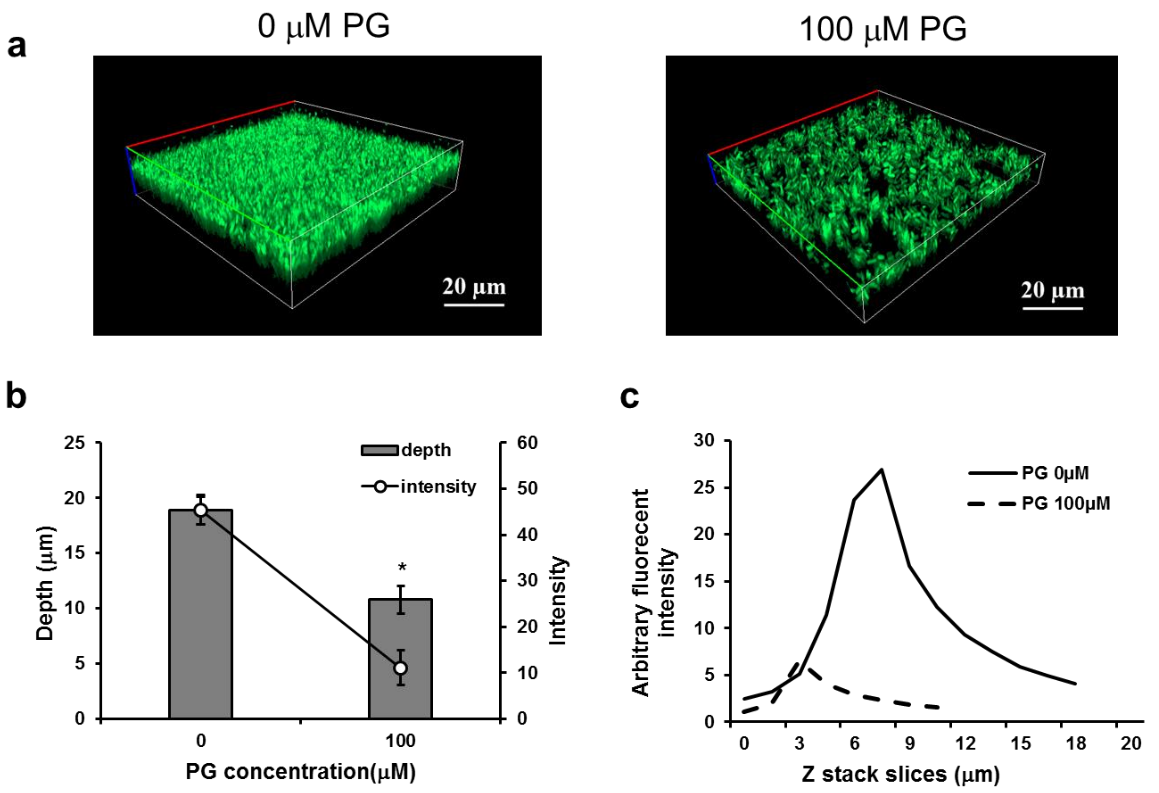

Supplementary Figure S4. Confocal laser scanning microscope analyses of *P. aeruginosa* biofilms treated with PG. (a) PAO1 cells were grown on glass coverslips in a 24-well plate for 6 h in the medium containing 0 μM or 100 μM PG. Biofilms were stained with the BacLight Live/Dead Viability Kit. Cells staining green are viable cells. The experiments were performed twice and representative images are shown. The scale bar represents 20 μm. (b) Biofilm thickness and quantification of green fluorescent intensities of two biofilms. Data represent the average of image stacks collected from five randomly selected areas. *, $P < 0.0001$ versus biofilm from untreated PAO1 cells. (c) The green fluorescent intensities in each of the sliced focal planes taken at 1.5 μm intervals are plotted as a function of biofilm height and compared between two biofilms.

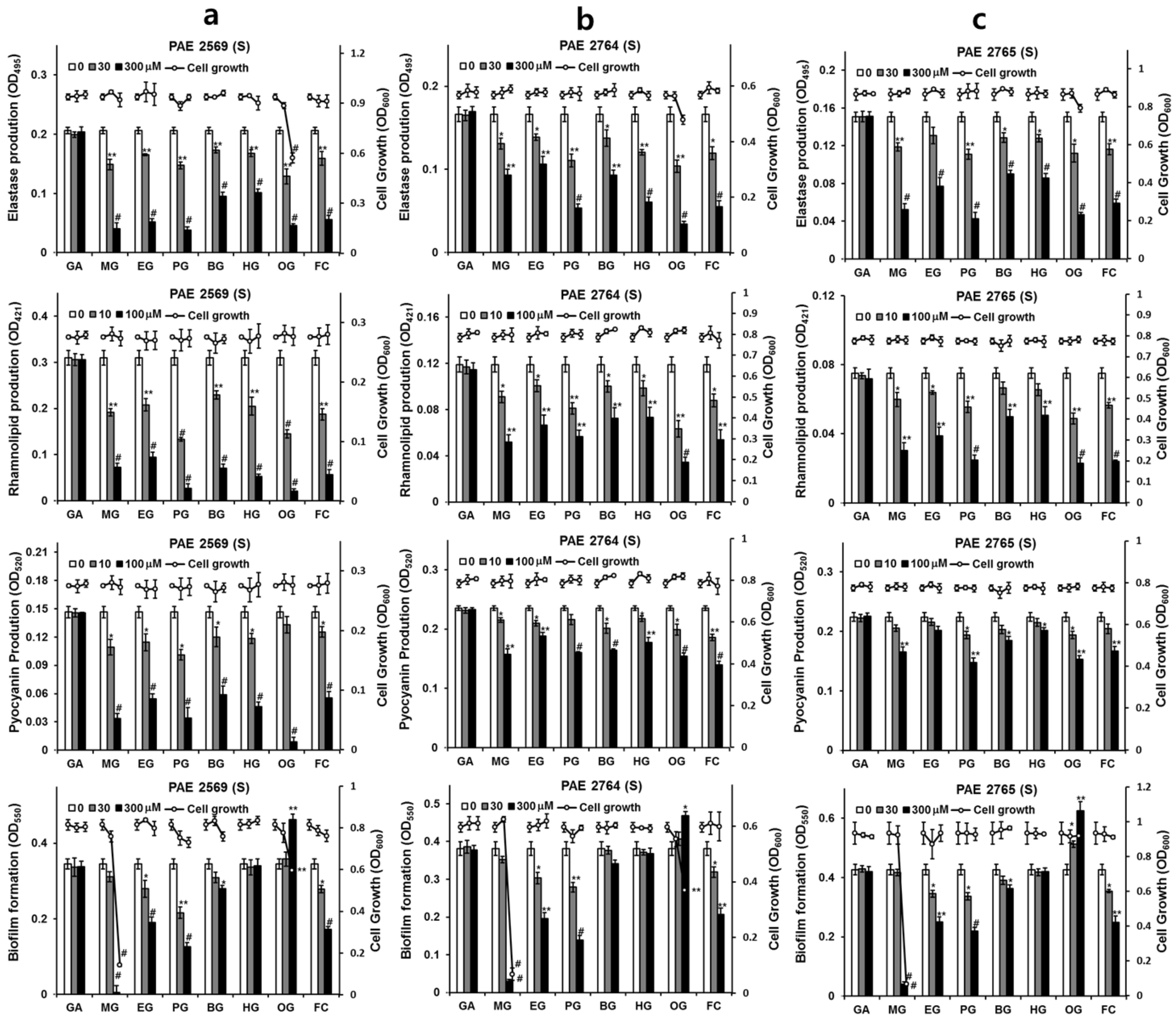

Supplementary Figure S5. The anti-virulence and anti-biofilm effects of the alkyl gallates against drug-sensitive *P. aeruginosa* clinical isolates. Three drug-sensitive *P. aeruginosa* clinical isolates, PAE 2569 (S) (**a**), PAE 2764 (S) (**b**), and PAE 2765 (S) (**c**), were grown in LB medium containing different concentrations of alkyl gallates for 24 h, followed by the measurement of cell density at 600 nm, elastase activity, pyocyanin, and rhamnolipid in the culture supernatants. Effects of alkyl gallates on biofilm formation and cell viability in the clinical isolates. *P. aeruginosa* biofilms formed in the presence of alkyl gallates for 9 h. The biofilm cells attached to the well surface were assayed using crystal violet staining. Planktonic cells density were measured at 600 nm. Three independent experiments were performed in triplicate, and the mean ± standard deviation (SD) values are displayed in each bar. *, $P < 0.01$; **, $P < 0.001$; $^{\#}$, $P < 0.0001$ versus DMSO treatment.

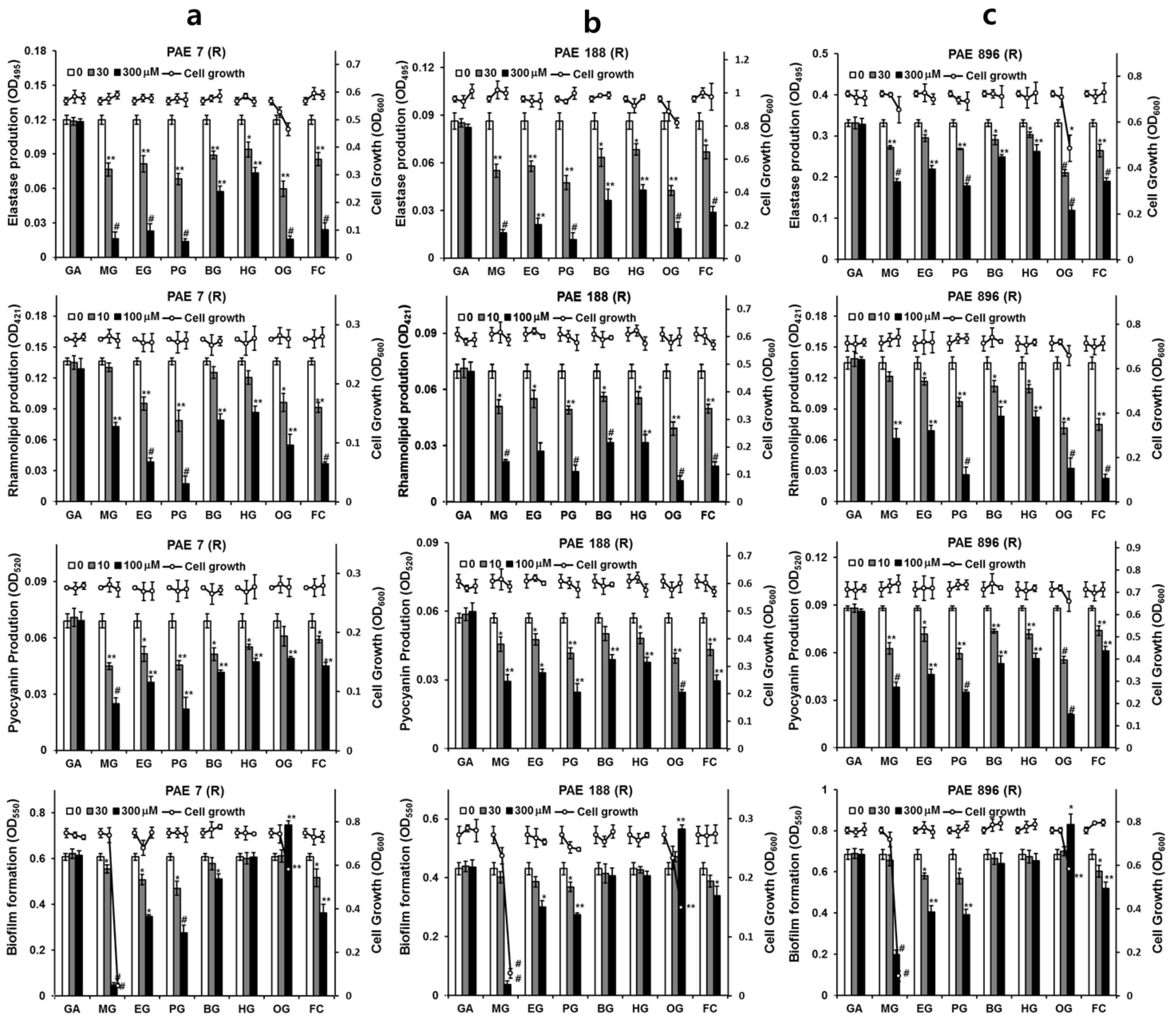

Supplementary Figure S6. The anti-virulence and anti-biofilm effects of the alkyl gallates against drug-resistant *P. aeruginosa* clinical isolates. (**a**, **b**, **c**) Three drug-resistant *P. aeruginosa* clinical isolates, PAE 7 (R), PAE 188 (R), and PAE 869 (R), were grown in LB medium containing different concentrations of alkyl gallates for 24 h, followed by the measurement of cell density at 600 nm, elastase activity, pyocyanin, and rhamnolipid in the culture supernatants. **(d)** Effects of alkyl gallates on biofilm formation and cell viability in the clinical isolates. *P. aeruginosa* biofilms formed in the presence of alkyl gallates for 9 h. The biofilm cells attached to the well surface were assayed using crystal violet staining. Planktonic cells density were measured at 600 nm. Three independent experiments were performed in triplicate, and the mean ± standard deviation (SD) values are displayed in each bar. *, $P < 0.01$; **, $P < 0.001$; $^{\#}$, $P < 0.0001$ versus DMSO treatment.

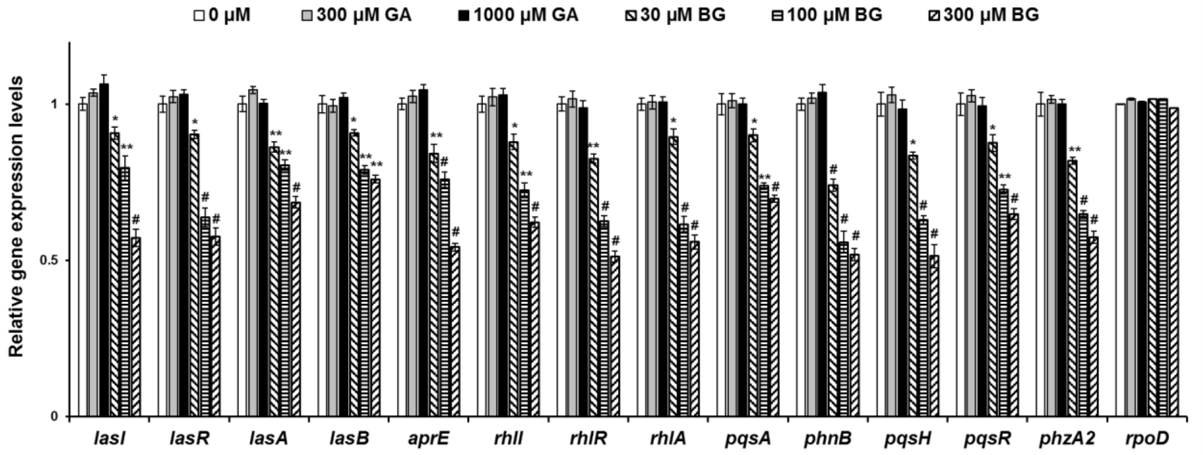

Supplementary Figure S7. Effect of free gallic acid (GA) and butyl gallate (BG) on the expression of QS-regulated genes in *P. aeruginosa* PAO1. PAO1 were cultured in LB medium containing with different GA or BG concentrations for 12 h. Effect of GA and BG on the expression of QS-regulated genes was assessed by RT-qPCR. The experiment shown is representative of three independent experiments in triplicate, and the mean ± SD values are displayed in each bar. *, $P < 0.01$; **, $P < 0.001$; $^{\#}$, $P < 0.0001$ versus DMSO treatment.

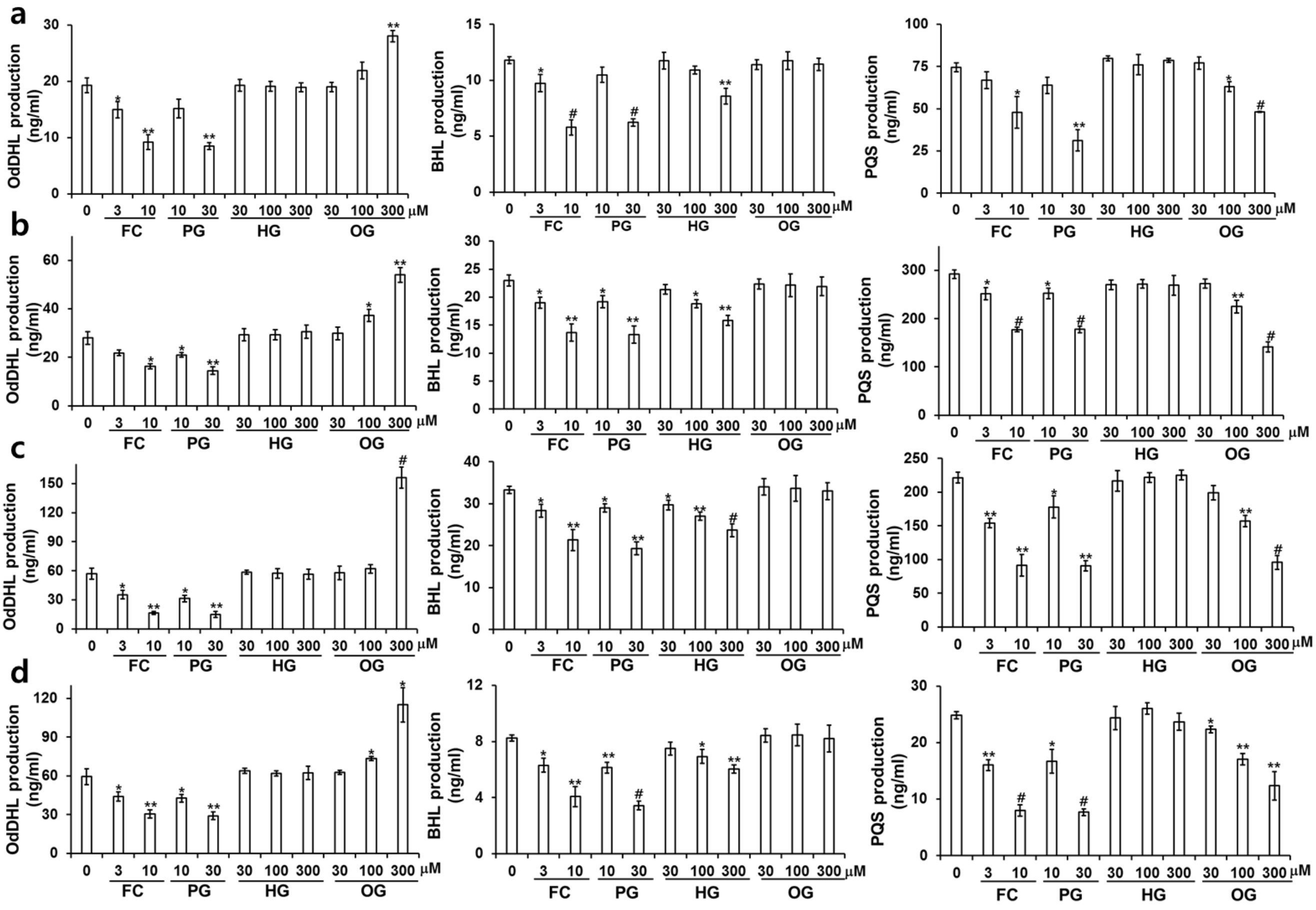

Supplementary Figure S8. Effects of alkyl gallates on QS signaling molecule production in clinical isolates of *P. aeruginosa*. Four clinical isolates, PAE 2569 (S) (**a**), PAE 2764 (S) (**b**), PAE 7 (R) (**c**), and PAE 188 (R) (**d**), were cultured in LB medium containing different alkyl gallates or furanone C-30 (FC) concentrations for 12 h. The three main QS molecules, OdDHL, BHL, and PQS, were extracted from the culture supernatants and quantitatively analyzed by LC-MS/MS. The experiment shown is representative of three independent experiments in triplicate, and the mean ± SD values are displayed in each bar. *, $P < 0.01$; **, $P < 0.001$; $^{\#}$, $P < 0.0001$ versus DMSO treatment.

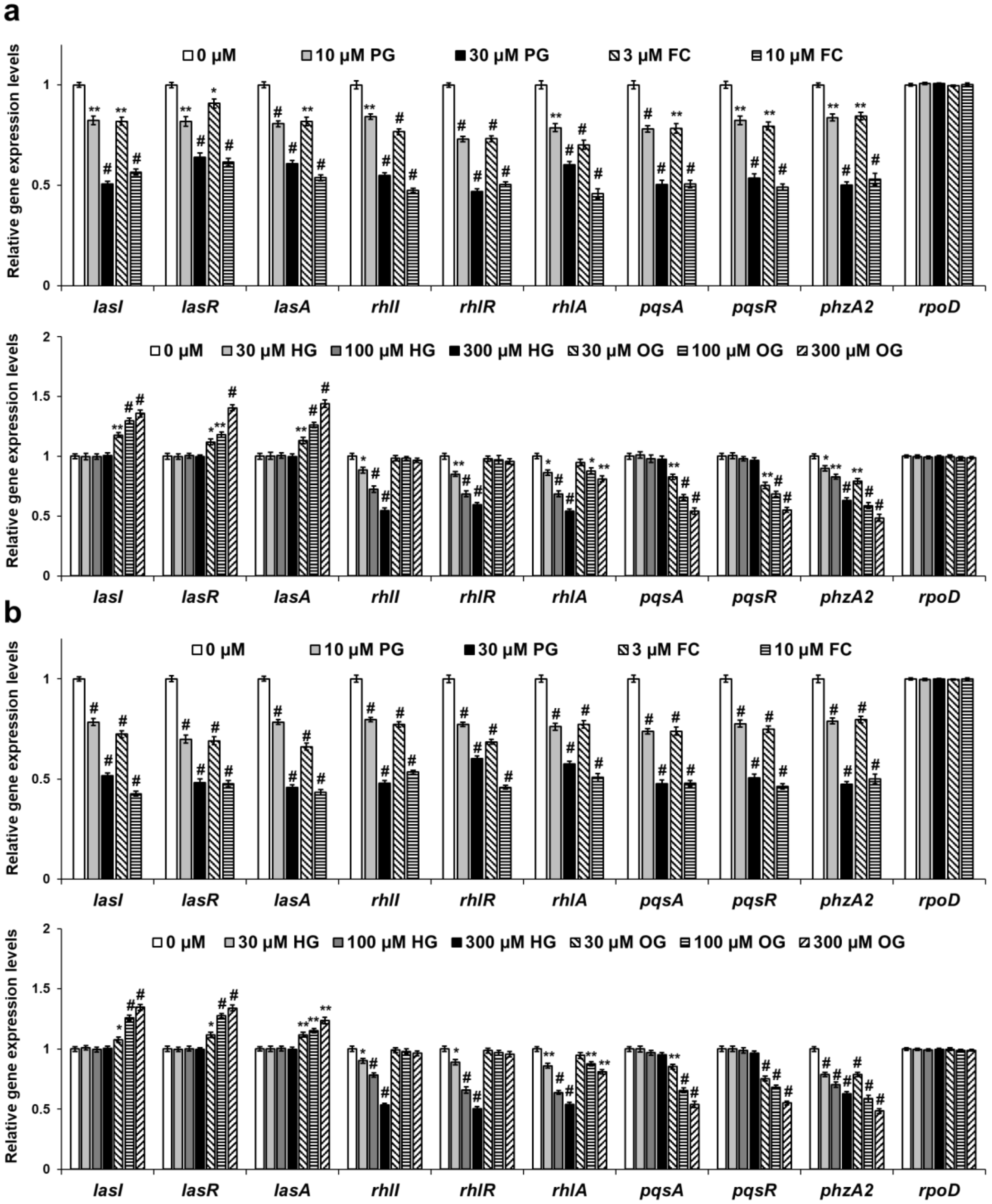

Supplementary Figure S9. Effects of alkyl gallates on QS gene expression in drug-sensitive clinical isolates of *P*. *aeruginosa*. Two drug-sensitive clinical isolates, PAE 2569 (S) (**a**) and PAE 2764 (S) (**b**) were cultured in LB medium containing different alkyl gallates or furanone C-30 (FC) concentrations for 12 h. Effect of alkyl gallates on the expression of QS-regulated genes were assessed by RT-qPCR. The experiment shown is representative of three independent experiments in triplicate, and the mean ± SD values are displayed in each bar. *, *P* < 0.01; **, *P* < 0.001; $^{\#}$, *P* < 0.0001 versus DMSO treatment.

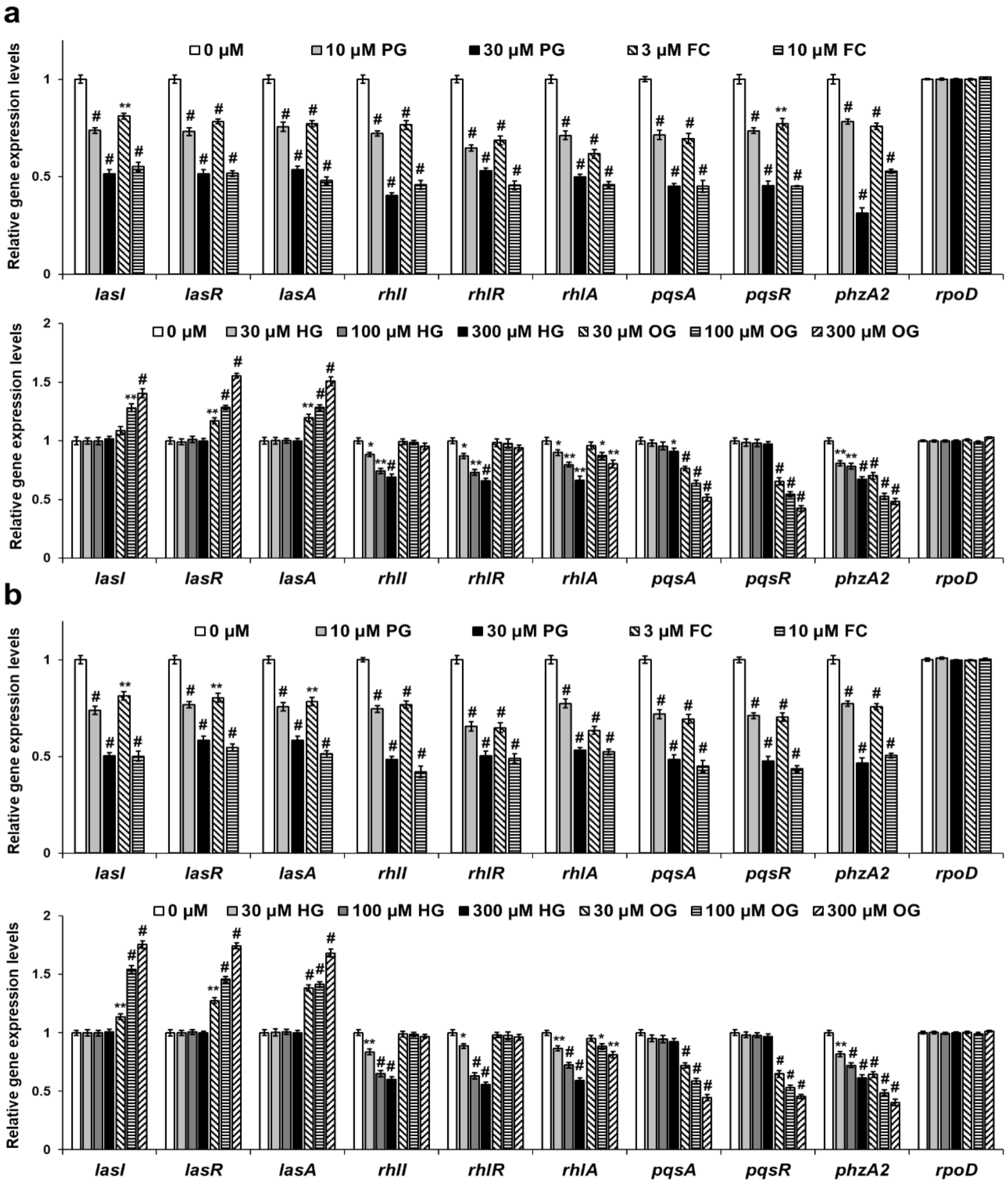

Supplementary Figure S10. Effects of alkyl gallates on QS gene expression in drug-resistant clinical isolates of *P. aeruginosa*. Two drug-resistant clinical isolates, PAE 7 (R) (**a**), and PAE 188 (R) (**b**), were cultured in LB medium containing different alkyl gallates or furanone C-30 (FC) concentrations for 12 h. Effect of alkyl gallates on the expression of QS-regulated genes were assessed by RT-qPCR. The experiment shown is representative of three independent experiments in triplicate, and the mean ± SD values are displayed in each bar. *, $P < 0.01$; **, $P < 0.001$; $^{\#}$, $P < 0.0001$ versus DMSO treatment.

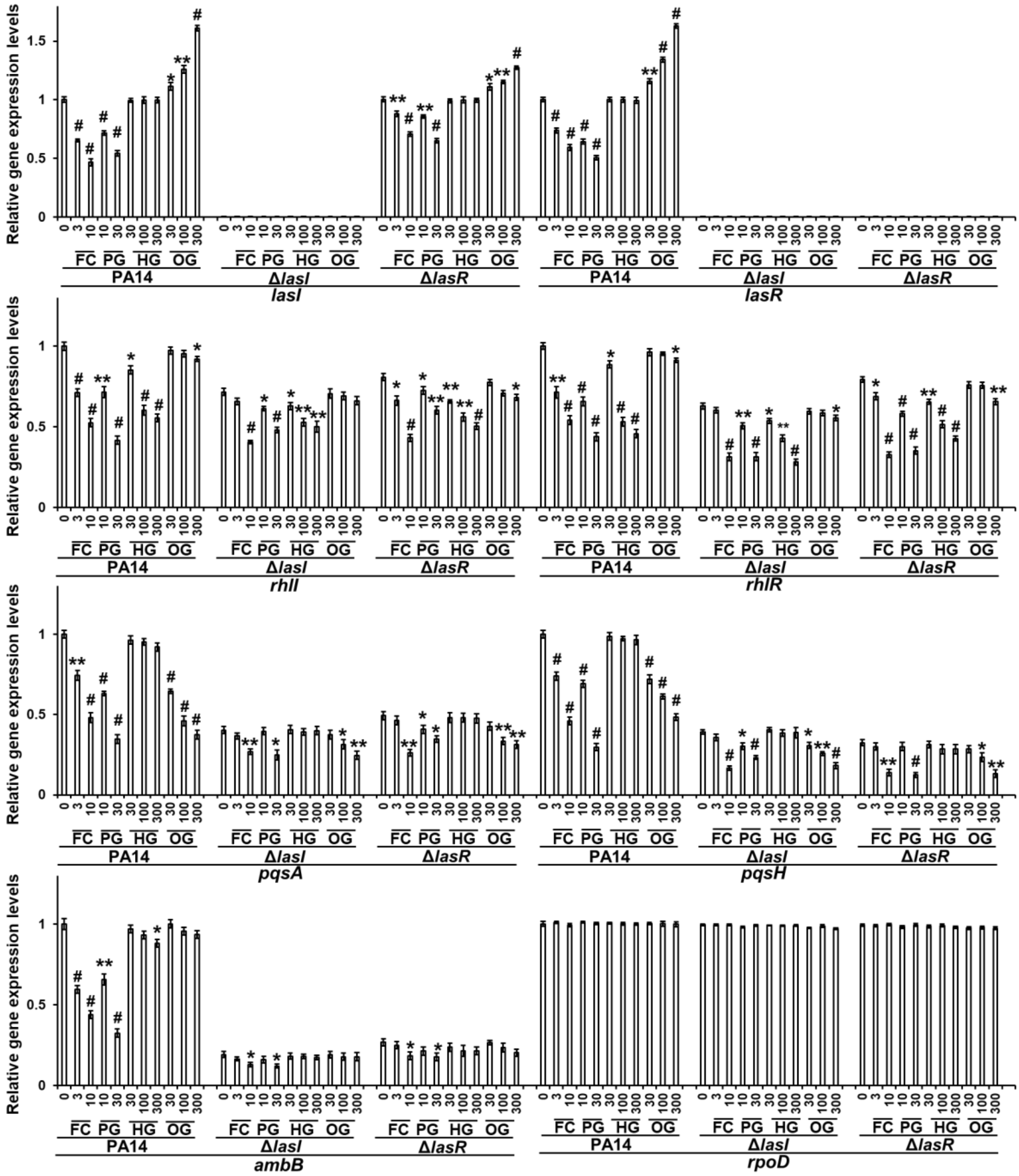

Supplementary Figure S11. Effects of alkyl gallates on IQS gene expression in the wild strain (PA14), Δ*las I* mutant, and Δ*lasR* mutant in low phosphate *Pseudomonas* medium supplemented with phosphate. The strains were cultured in low phosphate *Pseudomonas* medium supplemented with 4 mM K_2HPO_4 containing different alkyl gallates or furanone C-30 (FC) concentrations for 12 h. Effect of alkyl gallates on the expression of IQS biosynthesis gene (*ambB*) including other QS-regulated genes were assessed by RT-qPCR. The experiment shown is representative of three independent experiments in triplicate, and the mean ± SD values are displayed in each bar. *, $P < 0.01$; **, $P < 0.001$; $^{\#}$, $P < 0.0001$ versus DMSO treatment.

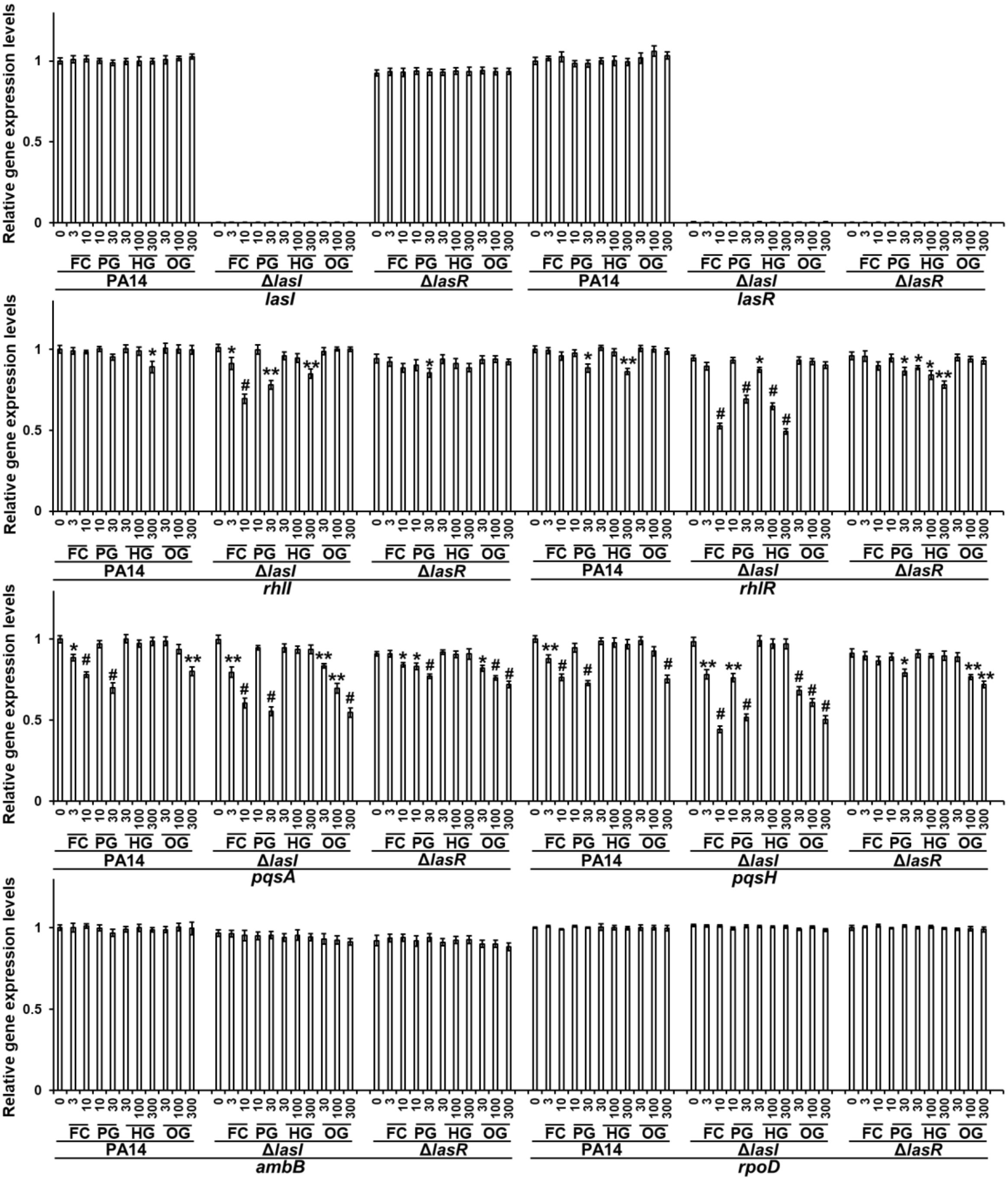

Supplementary Figure S12. Effects of alkyl gallates on IQS gene expression in the wild strain (PA14), Δ*las I* mutant, and Δ*lasR* mutant in low phosphate *Pseudomonas* medium. The strains were cultured in low phosphate *Pseudomonas* medium containing different alkyl gallates or furanone C-30 (FC) concentrations for 12 h. Effect of alkyl gallates on the expression of IQS biosynthesis gene (*ambB*) including other QS-regulated genes were assessed by RT-qPCR. The experiment shown is representative of three independent experiments in triplicate, and the mean ± SD values are displayed in each bar. *, $P < 0.01$; **, $P < 0.001$; $^{\#}$, $P < 0.0001$ versus DMSO treatment.

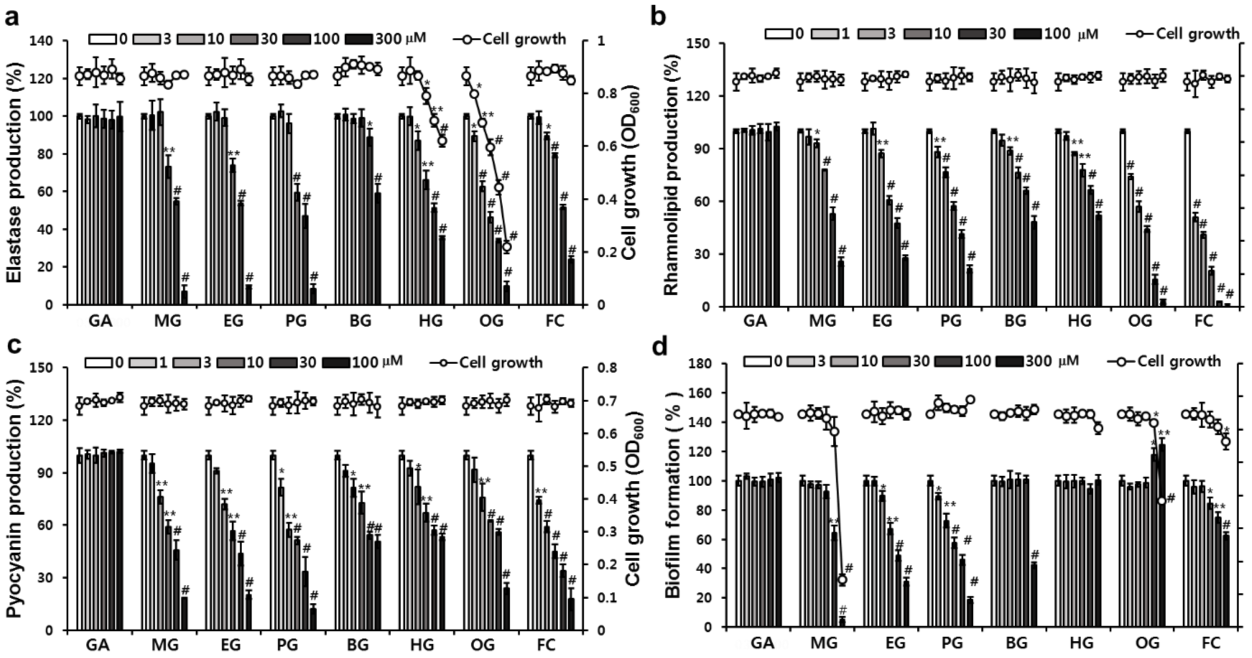

Supplementary Figure S13. Effect of alkyl gallates on virulence factor production and biofilm formation in *P. aeruginosa* PA14. **(a, b, c)** PA14 cells were grown in LB medium containing different concentrations of alkyl gallates for 24 h, followed by the measurement of cell density at 600 nm, elastase activity, pyocyanin, and rhamnolipid in the culture supernatants. **(d)** Effects of alkyl gallates on biofilm formation and cell viability in *P. aeruginosa*. *P. aeruginosa* biofilms formed in the presence of alkyl gallates for 9 h. The biofilm cells attached to the well surface were assayed using crystal violet staining. Planktonic cells density were measured at 600 nm. Three independent experiments were performed in triplicate, and the mean ± standard deviation (SD) values are displayed in each bar. *, $P < 0.01$; **, $P < 0.001$; $^{\#}$, $P < 0.0001$ versus DMSO treatment.

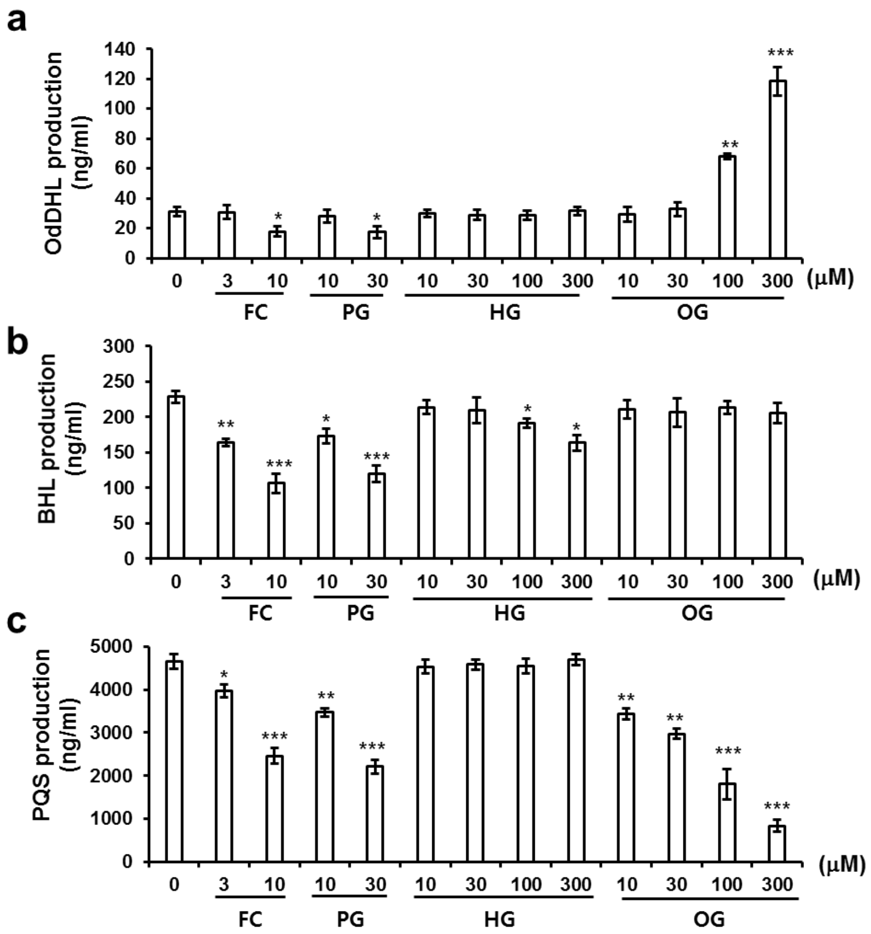

Supplementary Figure S14. Effects of alkyl gallates on QS signaling molecule production in *P. aeruginosa* PA14. PA14 cells were cultured in LB medium containing different alkyl gallates or furanone C-30 (FC) concentrations for 12 h. The three main QS molecules, OdDHL (**a**), BHL (**b**), and PQS (**c**), were extracted from the culture supernatants and quantitatively analyzed by LC-MS/MS. The experiment shown is representative of three independent experiments in triplicate, and the mean ± SD values are displayed in each bar. *, $P < 0.01$; **, $P < 0.001$; ***, $P < 0.0001$ versus DMSO treatment.

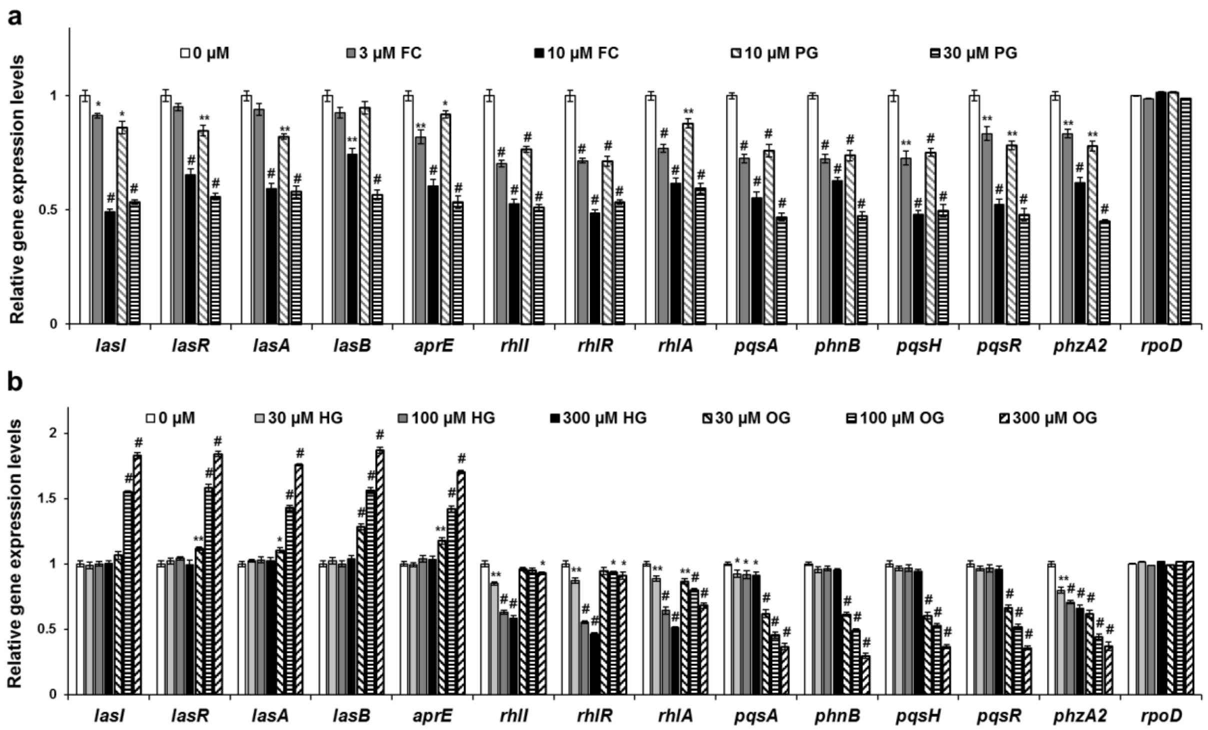

Supplementary Figure S15. Effects of alkyl gallates on QS gene expression in *P. aeruginosa* PA14. PA14 cells were cultured in LB medium containing different alkyl gallates or furanone C-30 (FC) concentrations for 12 h. Effect of alkyl gallates on the expression of QS-regulated genes were assessed by RT-qPCR. The experiment shown is representative of three independent experiments in triplicate, and the mean ± SD values are displayed in each bar. *, $P < 0.01$; **, $P < 0.001$; $^{\#}$, $P < 0.0001$ versus DMSO treatment.

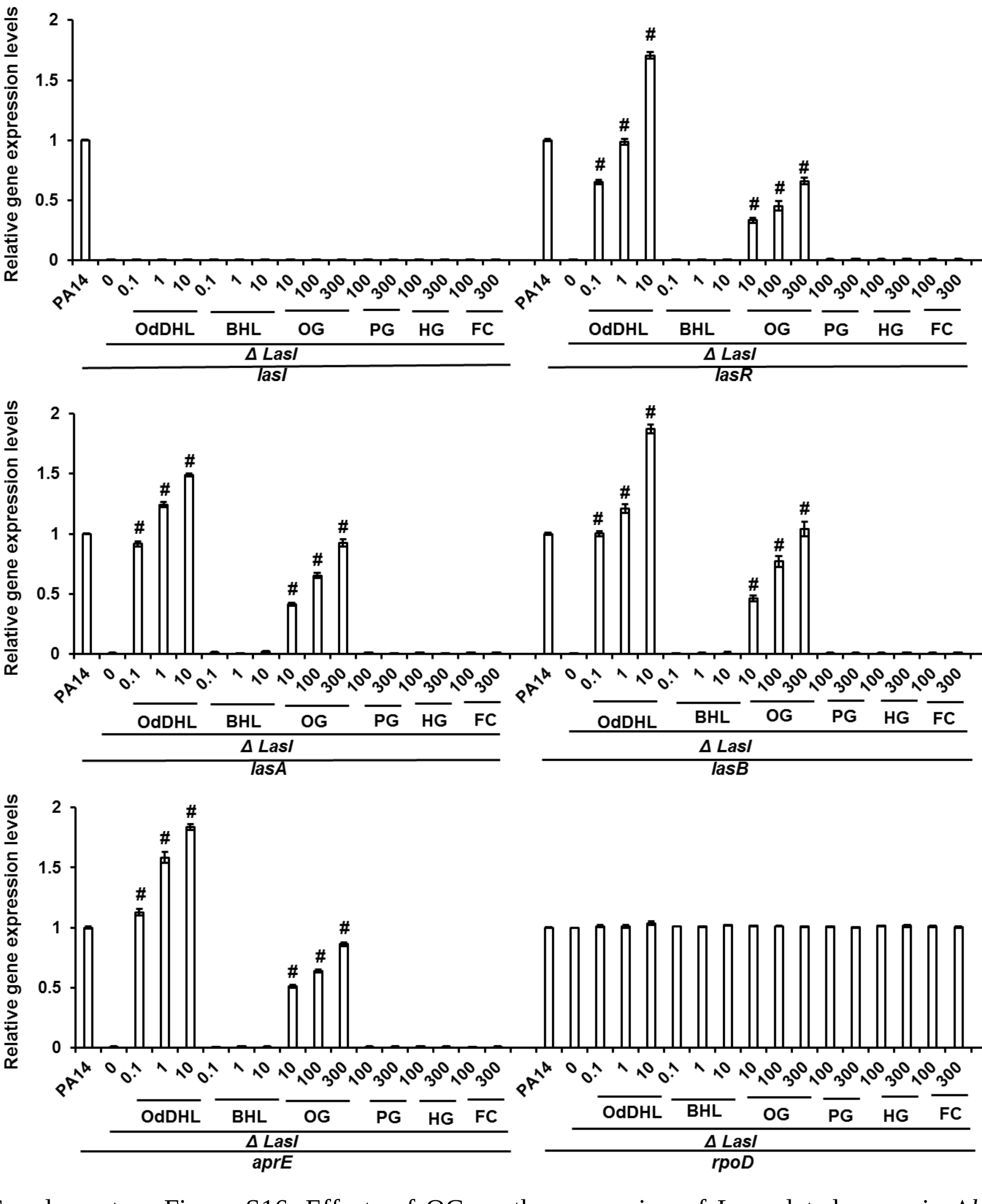

Supplementary Figure S16. Effects of OG on the expression of Las-related genes in Δ*lasI* mutant. The ΔlasI mutant of PA14 were cultured in medium containing different concentrations of alkyl gallates and QS ligands for 12 h. Effect of alkyl gallates on the expression of Las-related genes were assessed by RT-qPCR. The experiment shown is representative of three independent experiments in triplicate, and the mean ± SD values are displayed in each bar. *, $P < 0.01$; **, $P < 0.001$; $^{\#}$, $P < 0.0001$ versus DMSO treatment.

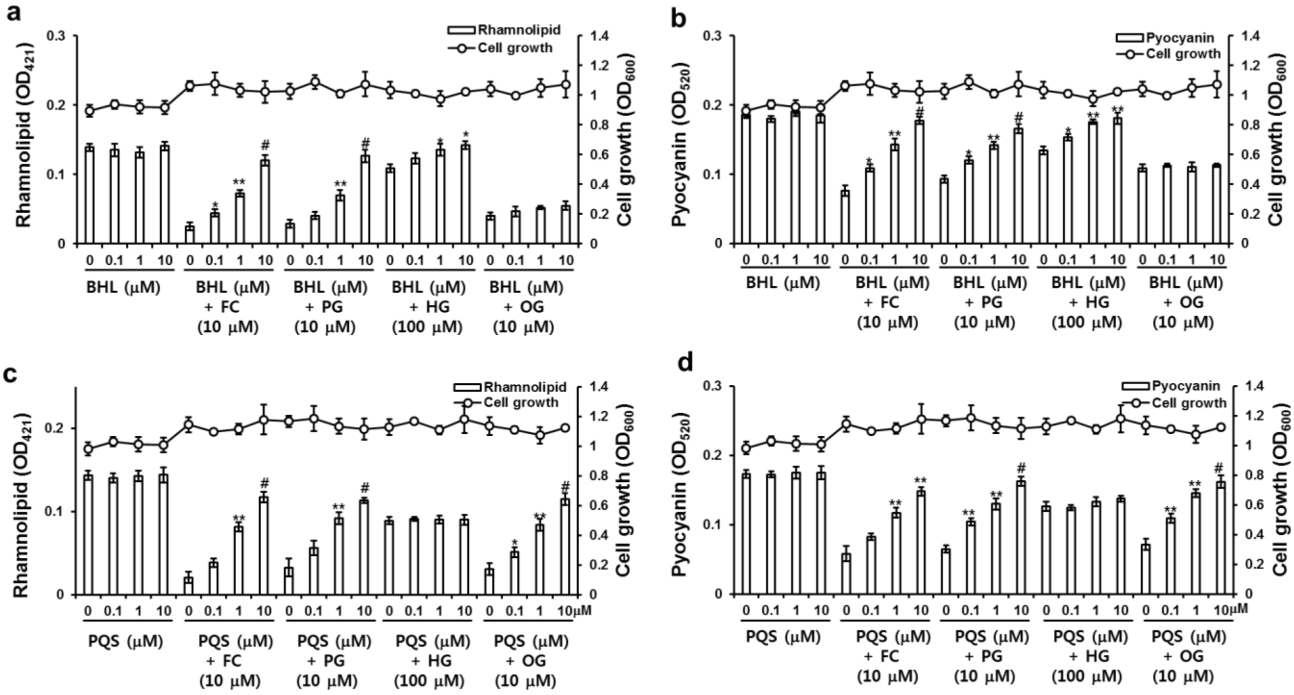

Supplementary Figure S17. Effects of exogenous QS ligands on HG- and OG-induced inhibition of rhamnolipid production in *P. aeruginosa* PA14. (**a** and **b**) Rhamnolipid production in PA14 cells cultured with alkyl gallates in the presence or absence of a different concentration of BHL (**a**) or PQS (**b**) for 18 h. (**c** and **d**) Pyocyanin production in PA14 cells cultured with alkyl gallates in the presence or absence of a different concentration of BHL (**c**) or PQS (**d**) for 18 h. The experiment shown is representative of three independent experiments in triplicate, and the mean ± SD values are displayed in each bar. *, $P < 0.01$; **, $P < 0.001$; $^{\#}$, $P < 0.0001$ versus DMSO treatment.

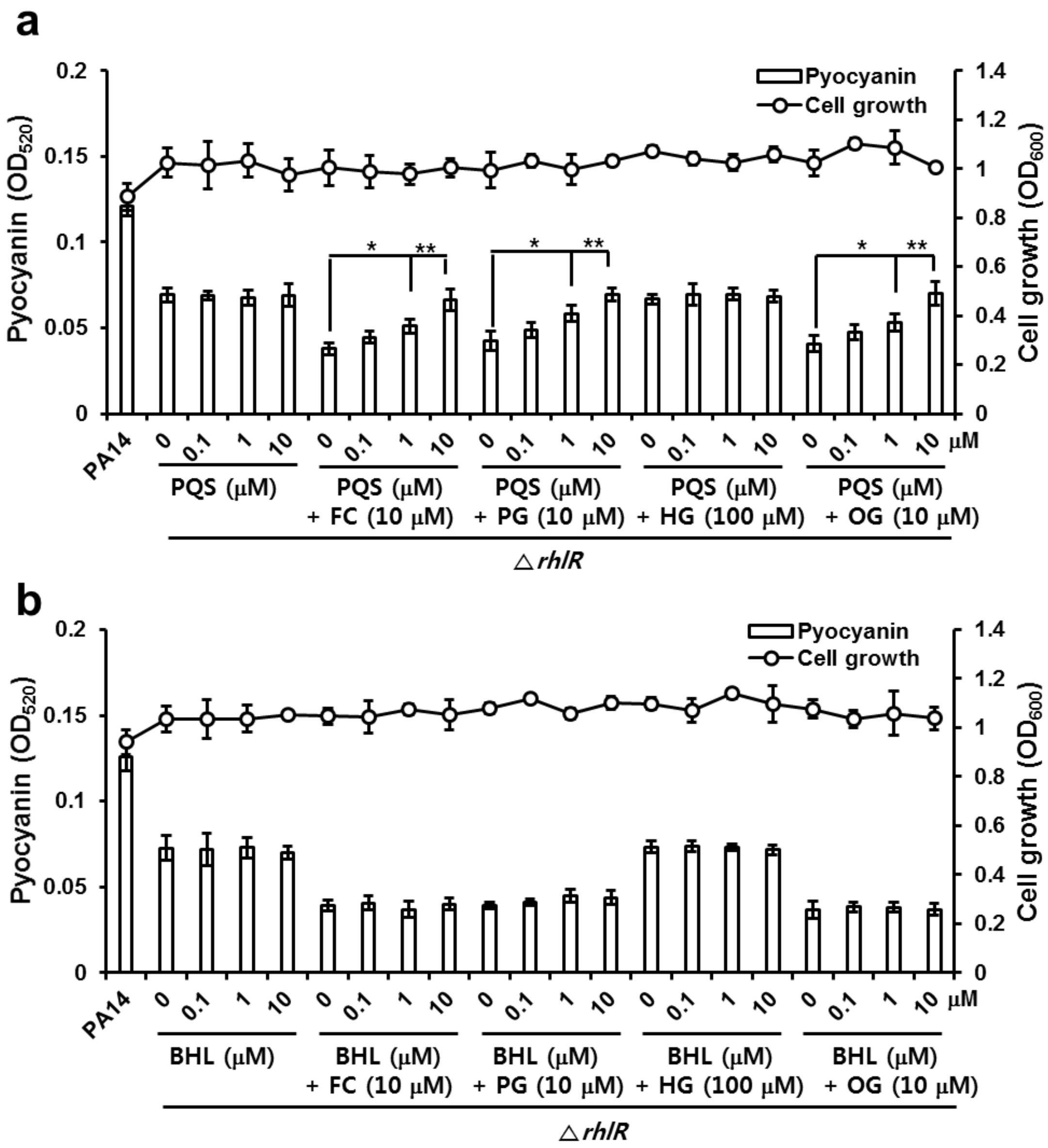

Supplementary Figure S18. Effects of exogenous QS ligands on HG- and OG-induced inhibition of pyocyanin production in Δ*rhlR* mutant. Pyocyanin production in the Δ*rhlR* mutant cultured with alkyl gallates in the presence or absence of a different concentration of PQS (**a**) or BHL (**b**) for 18 h. The experiment shown is representative of three independent experiments in triplicate, and the mean ± SD values are displayed in each bar. *, $P < 0.01$; **, $P < 0.001$ versus DMSO treatment.

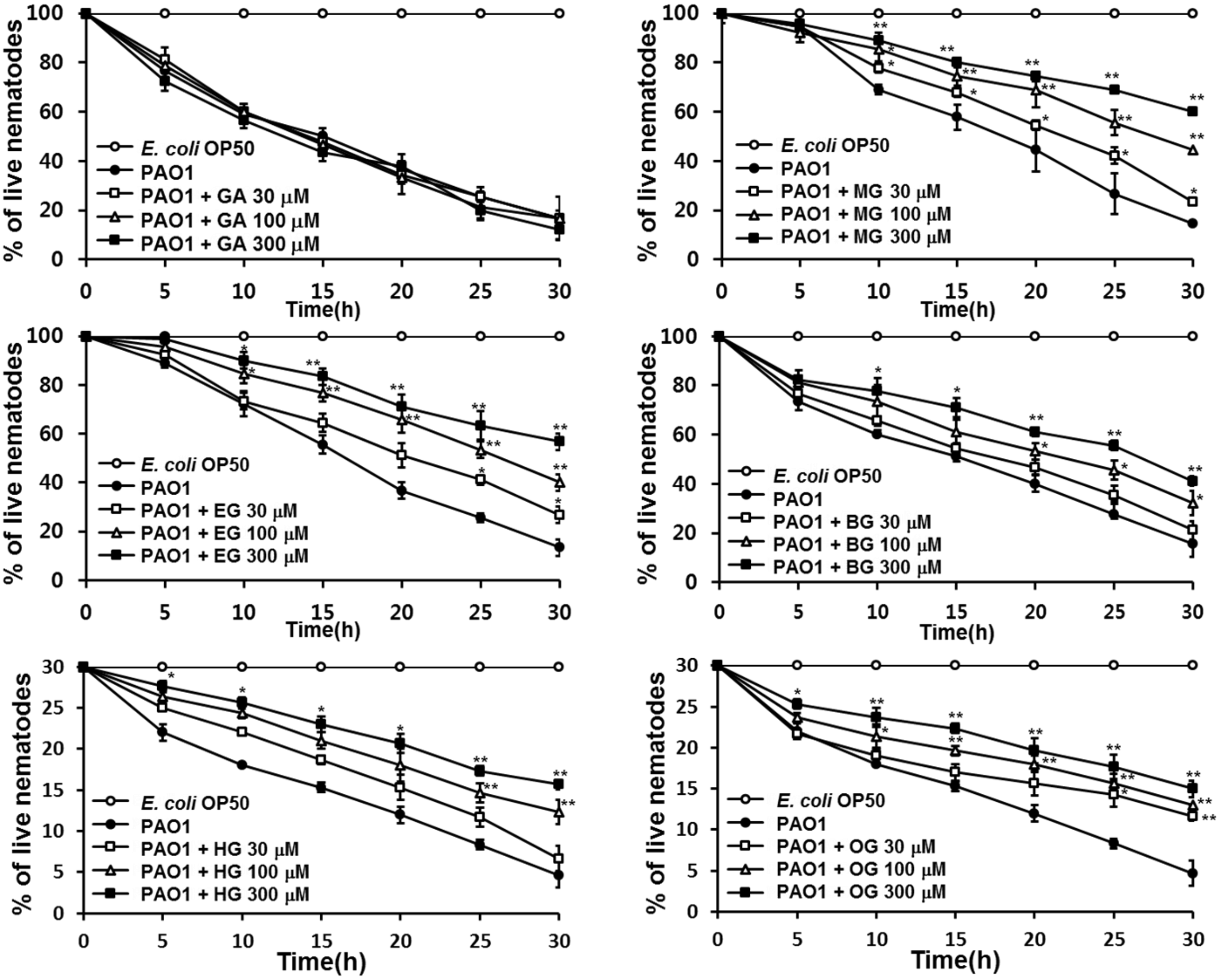

Supplementary Figure S19. Inhibition of PAO1 virulence toward *C. elegans* by other alkyl gallates. Thirty worms were introduced on lawns of *E. coli* OP50 (open circles) or PAO1 (filled circles) on plates containing different concentrations of alkyl gallates. The percentage of live nematodes was calculated every 5 h for 30 h. Three independent experiments were performed in triplicate, and the mean ± SD values are presented in each graph. *, $P < 0.01$; **, $P < 0.001$ versus untreated cells. .

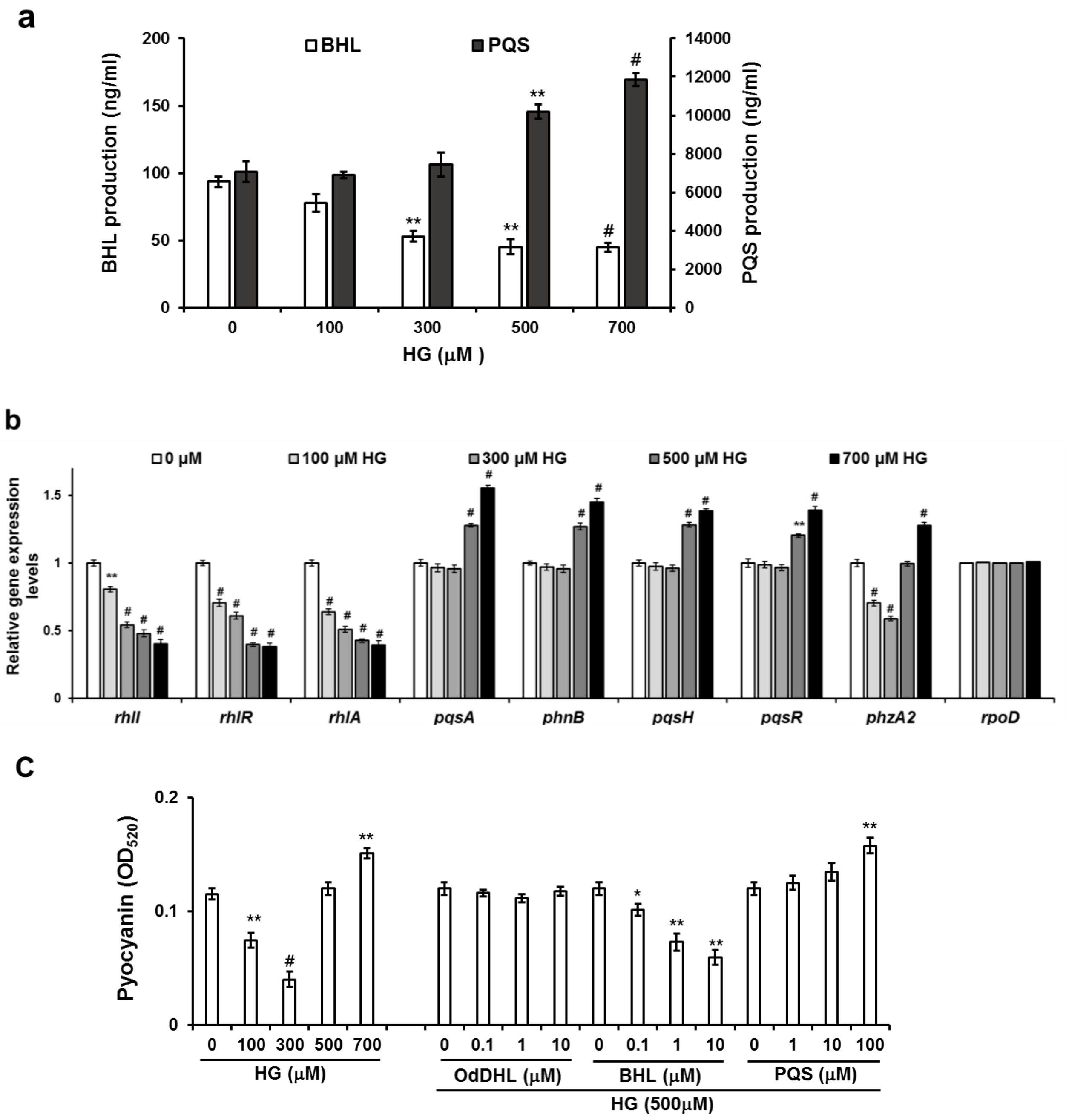

Supplementary Figure S20. Effects of higher concentrations of HG on Pqs QS system in *P. aeruginosa* PA14. (**a** and **b**) Effects of higher concentration of HG (500 and 700 μM) on PQS signal molecule production and the expression of pqs-related gens. (**c**) Higher concentrations of HG (500 and 700 μM)-induced enhancement of pyocyanin and reversion of the HG (500 μM)-induced enhancement of pyocyanin by exogenous BHL. The experiment shown is representative of three independent experiments in triplicate, and the mean ± SD values are displayed in each bar. *, $P < 0.01$; **, $P < 0.001$; #, $P < 0.0001$ versus DMSO treatment.
